# *ELOVL5* and *IGFBP6* genes modulate sensitivity of breast cancer cells to ferroptosis

**DOI:** 10.3389/fmolb.2023.1075704

**Published:** 2023-01-13

**Authors:** Sergey Nikulin, Alexandra Razumovskaya, Andrey Poloznikov, Galina Zakharova, Boris Alekseev, Alexander Tonevitsky

**Affiliations:** ^1^ Faculty of Biology and Biotechnologies, Higher School of Economics, Moscow, Russia; ^2^ P. A. Hertsen Moscow Oncology Research Center, Branch of the National Medical Research Radiological Center, Ministry of Health of the Russian Federation, Moscow, Russia; ^3^ World-Class Research Center “Digital Biodesign and Personalized Healthcare”, Sechenov First Moscow State Medical University, Moscow, Russia; ^4^ Shemyakin-Ovchinnikov Institute of Bioorganic Chemistry, Russian Academy of Sciences, Moscow, Russia

**Keywords:** breast cancer, ELOVL5, IGFBP6, PUFA, ferroptosis

## Abstract

**Introduction:** Relapse of breast cancer is one of the key obstacles to successful treatment. Previously we have shown that low expression of *ELOVL5* and *IGFBP6* genes in breast cancer tissue corresponded to poor prognosis. *ELOVL5* participates directly in the elongation of polyunsaturated fatty acids (PUFAs) that are considered to play an important role in cancer cell metabolism. Thus, in this work we studied the changes in lipid metabolism in breast cancer cells with reduced expression of either *ELOVL5* or *IGFBP6* gene.

**Methods:** MDA-MB-231 cells with a stable knockdown of either *ELOVL5* or *IGFBP6* gene were used in this study. Transcriptomic and proteomic analysis as well as RT-PCR were utilized to assess gene expression. Content of individual fatty acids in the cells was measured with HPLC-MS. HPLC was used for analysis of the kinetics of PUFAs uptake. Cell viability was measured with MTS assay. Flow cytometry was used to measure activation of apoptosis. Fluorescent microscopy was utilized to assess accumulation of ROS and formation of lipid droplets. Glutathione peroxidase activity was measured with a colorimetric assay.

**Results:** We found that the knockdown of IGFBP6 gene led to significant changes in the profile of fatty acids in the cells and in the expression of many genes associated with lipid metabolism. As some PUFAs are known to inhibit proliferation and cause death of cancer cells, we also tested the response of the cells to single PUFAs and to combinations of docosahexaenoic acid (DHA, a n-3 PUFA) with standard chemotherapeutic drugs. Our data suggest that external PUFAs cause cell death by activation of ferroptosis, an iron-dependent mechanism of cell death with excessive lipid peroxidation. Moreover, both knockdowns increased cells’ sensitivity to ferroptosis, probably due to a significant decrease in the activity of the antioxidant enzyme GPX4. Addition of DHA to commonly used chemotherapeutic drugs enhanced their effect significantly, especially for the cells with low expression of *IGFBP6* gene.

**Discussion:** The results of this study suggest that addition of PUFAs to the treatment regimen for the patients with low expression of *IGFBP6* and *ELOVL5* genes can be potentially beneficial and is worth testing in a clinically relevant setting.

## 1 Introduction

Breast cancer (BC) continues to remain the leading cause of morbidity and mortality of women from malignant neoplasms in the world (Sung et al., 2021). Recurrence of BC happens in around 40% of the patients (Gerber et al., 2010; Lafourcade et al., 2018). One of the promising treatment strategies relies on identifying patients with high risk of recurrence with the help of gene-expression signatures in the early stages of the disease and intensifying treatment regimen for them (Kwa et al., 2017). Previously, our group reported a new method for predicting outcomes based on prognostic markers, in which the expression levels of two genes: Insulin Like Growth Factor Binding Protein 6 (*IGFBP6*) and Elongation Of Very Long chain fatty acids protein 5 (*ELOVL5*) made it possible to predict breast cancer recurrence in the first 5 years of follow-up with high sensitivity (81.8%) and specificity (62.5%), while high expression of *ELOVL5* and *IGFBP6* corresponded to a favorable prognosis (Galatenko et al., 2015).

*ELOVL5* is a human elongase of long-chain polyunsaturated fatty acids (LC-PUFAs) located in the endoplasmic reticulum membrane (Leonard et al., 2000; Wang et al., 2008; Moon et al., 2009). It is known that n-3 and n-6 polyunsaturated fatty acids can affect the development of breast cancer, and in some cases they can cause death of breast cancer cells (Liput et al., 2021). Moreover, polyunsaturated fatty acids alter breast cancer cell adhesion and metastasis in n-6 and n-3 PUFA-treated nude mice and affect mRNA expression in breast cancer cells that encode metastasis-associated metalloproteinases (Johanning and Lin, 1995).

On the other hand, *IGFBP6* is a secreted protein that binds to insulin-like growth factors (*IGFs*), preventing their action on cells (Bach et al., 2013). Particular attention was paid to the study of the role of IGFBP6 in tumor metastasis. For example, it was shown that the expression level of IGFBP6 in colon cancer cells with a high metastatic potential is lower than in the cells with a low metastatic potential, and the expression of IGFBP6 in secondary squamous cell carcinomas of the head and neck is lower than in primary ones, which indirectly indicates that that IGFBP6 reduces the metastatic potential of tumor cells (Bach, 2015). Acting as a transcription factor, IGFBP6 is able to enter the nucleus through the NLS sequence and importin-α (Bach, 2016). In the nucleus, IGFBP6 binds to the EGR-1 gene promoter and stimulates its expression. Expression of EGR1 leads to inhibition of migration and invasion, as well as inhibition of proliferation and triggering of apoptosis.

IGFBP6 can also reduce cell viability through other IGF-independent mechanisms. In particular, it is known that IGFBP6 locks the Ku80 protein in the cytosol, preventing it from entering the nucleus, where it is involved in the repair of DNA double-strand breaks (Bach, 2016). In turn, the accumulation of DNA defects promotes apoptosis. Finally, a recent study identified a relationship between the GPR81 lactate receptor and the IGFBP6 protein, which is able to modulate lactate metabolism and oxidative stress in human MDA-MB-231 cells, thus influencing breast cancer growth (Longhitano et al., 2022).

Recently we have found that knockdown of either *ELOVL5* or *IGFBP6* in breast cancer cell line MDA-MB-231 led to a strong increase in the expression of the matrix metalloproteinase (MMP) *MMP1*, as well as to a change in the expression of a group of genes involved in the formation of intercellular contacts (Nikulin et al., 2021). Analysis of spheroid formation confirmed that intercellular adhesion decreased as a result of both *ELOVL5* and *IGFBP6* knockdowns, thus suggesting that malignant breast tumors with reduced expression of *ELOVL5* or *IGFBP6* gene may metastasize more actively due to more efficient tumor cell invasion (Nikulin et al., 2021).

In addition to the changes in cell adhesion, we also noted changes in the fatty acid metabolism pathway for both knockdown lines (Nikulin et al., 2021). *ELOVL5* is directly involved in LC-PUFAs’ metabolism and changes in fatty acids composition were expected for cells with *ELOVL5* knockdown. However, to our surprise, the decrease in *IGFBP6* expression also altered PUFAs’ profile (Nikulin et al., 2021).

It is well known that PUFAs can affect the behavior of cancer cells. In particular, n-3 and n-6 LC-PUFAs change gene expression profile in tumor cells in different ways (Hammamieh et al., 2007). A number of studies have also shown that introduction of n-3 LC-PUFAs into the culture medium inhibits proliferation, migration, and invasion of tumor cells (Chamras et al., 2002; Ding et al., 2013; Huang et al., 2017). Data on the effects of n-6 LC-PUFAs on cancer cells are contradictory. Different studies reported that inhibitory effects of n-6 LC-PUFAs on tumor cells are less pronounced than those of n-3 LC-PUFAs, absent altogether, and even found stimulating effects of n-6 LC-PUFAs on tumor cells (Connolly and Rose, 1993; Schley et al., 2005; Gonzalez-Reyes et al., 2017).

One of the effects of LC-PUFAs on cancer cells that can potentially be utilized for therapy is induction of ferroptotic cell death (Chen et al., 2021a; Dierge et al., 2021). Ferroptosis is an iron-dependent, non-apoptotic mode of cell death characterized by the accumulation of lipid-reactive oxygen species (ROS). Ferroptosis does not have the morphological features of typical necrosis, such as swelling of the cytoplasm and organelles, and rupture of the cell membrane, nor does it have the characteristics of traditional cellular apoptosis, such as cell shrinkage, chromatin condensation, formation of apoptotic bodies, and disintegration of the cytoskeleton. And unlike in autophagy, the formation of classical closed bilayer membrane structures (autophagic vacuoles) does not occur during ferroptosis (Li et al., 2020). Morphologically, ferroptosis is mainly manifested in the apparent contraction of mitochondria with increased membrane density and reduction or disappearance of mitochondrial cristae, which differs from other modes of cell death (Dixon et al., 2012).

PUFAs peroxidation in cell membrane leads to disruption of cell integrity. Long-term excessive accumulation of fatty acids from the environment medium triggers ferroptosis (Chen et al., 2021a; Dierge et al., 2021). Ferroptosis can also be induced by inhibition of antioxidants (GPx4) that normally prevent lipid peroxidation (Seibt et al., 2019). During lipid peroxidation free radicals predominantly attack PUFAs. This fact is explained by the greatest susceptibility of multiple double bonds to peroxidation. (Dierge et al., 2021).

In this study, we focused on the role of *ELOVL5* and *IGFBP6* genes in the metabolism of LC-PUFAs in breast cancer cells. We also assessed the influence of *ELOVL5* and *IGFBP6* gene knockdowns on ferroptosis induction and on cell response to excess of various LC-PUFAs. For this study we chose 2 shorter PUFAs (n-6 linoleic acid and n-3 α-linolenic acid) and 2 longer ones (n-6 arachidonic acid and n-3 docosahexaenoic acid) as their impact on breast cancer has been extensively studied previously in different setups (Banni, 1999; Bocca et al., 2008; Yee et al., 2010; Zhou et al., 2016; Geng et al., 2017; Huang et al., 2022). And finally, we tested how the knockdowns of *ELOVL5* and *IGFBP6* affect the cell response to standard of care (SOC) chemotherapeutics when they are combined with ferroptosis inducers.

## 2 Materials and methods

### 2.1 Cell culture

Stable knockdowns of *ELOVL5* and *IGFBP6* genes were performed using RNA interference (Schwankhaus et al., 2014; Nikulin et al., 2018; 2021; Maltseva et al., 2020). DNA oligonucleotides selected for the target sequences in the *ELOVL5* and *IGFBP6* genes were ligated into the pLVX shRNA1 lentiviral vector (Clontech Laboratories) according to the manufacturer’s protocol. To obtain the control MDA-MB-231 cells, we used the same lentiviral vector pLVX shRNA1 containing shRNA to the *Photinus pyralis* firefly luciferase gene. Viral particles were obtained in the form of cell-free supernatants using transient transfection of HEK-293T cell line according to the previously described method (Weber et al., 2010; 2012). Supernatants were collected 24 h after transfection, filtered using .45 µm syringe filters and stored at −80°C. Then, 5∙10^4^ MDA-MB-231 cells were cultured in the wells of a 24-well culture plate in 0.5 mL of cell culture medium. After 24 h, 10 μL of the supernatant containing viral particles was added to the wells, and the plate was placed in a cell culture incubator for 24 h. Then the cell culture medium was changed and the cells were incubated for another 24 h. After that, the selection with 1 μg/mL puromycin (Gibco) was carried out for 2 weeks.

Cells were cultured in a complete culture medium consisting of DMEM 4.5 g/L glucose (Gibco) supplemented with 10% vol. fetal bovine serum (Gibco) and 1% vol. antibiotic-antimycotic solution (Gibco). The cells were incubated in a cell culture incubator at 37°C, 5% CO_2_ (Sanyo). Subcultivation was performed every 2–3 days using trypsin-EDTA solution (PanEco). Cells were counted after trypan blue (Gibco) staining using Countess automated cell counter (Invitrogen) according to the manufacturer’s protocol.

### 2.2 Analysis of transcriptomic and proteomic data

We analyzed further the transcriptomic data that we have previously published (Nikulin et al., 2021) and deposited it as GSE165854 dataset. GSE165854 contains Human Transcriptome Array 2.0 microarray (Affymetrix) data for MDA-MB-231 cells with shRNA mediated knockdown of either *ELOVL5* or *IGFBP6* genes and for control MDA-MB-231(LUC) cells. ANOVA with Benjamin-Hochberg correction for multiple comparisons were used to access statistical significance of the differences observed between these cell lines.

The following datasets (Supplementary Table S1) from GEO (Gene Expression Omnibus) were used for correlation analysis: GSE102484 (Cheng et al., 2017), GSE22220 (Camps et al., 2008), GSE3494 (Miller et al., 2005), GSE58644 (Miller et al., 2005), GSE6532 (Loi et al., 2008). We also used data obtained by the METABRIC consortium (Cerami et al., 2012; Curtis et al., 2012) and The Cancer Genome Atlas (TCGA) program (Weinstein et al., 2013).

TAC 4.0 software (Thermo Fisher Scientific) was applied to preprocess raw data from Affymetrix microarrays. To carry out correlation analysis and statistical data processing, we employed the R 4.1 programming language with the RStudio 1.4 integrated development environment. The values of the Pearson correlation coefficient *R* and the *p*-values (the significance of the difference of *R* from zero) were calculated using the “cor.test” function. Correction for multiple comparisons was performed with the Benjamini-Hochberg method. The correlation coefficients with *p*-values less than .05 were considered significant.

Proteomic data from PXD023892 dataset was analyzed as well. NanoHPLC-MS/MS system coupled with a Q Exactive Orbitrap mass spectrometer (Thermo Fisher Scientific) was utilized to measure expression of proteins in the cells. Student’s *t*-test with Benjamin-Hochberg correction for multiple comparisons were used to access statistical significance of the observed changes. Detailed procedure was described earlier (Nikulin et al., 2021).

### 2.3 RT-PCR

Real-time PCR was used to confirm the changes in the expression of individual genes. RNA isolation was performed using miRNeasy Micro Kit (Qiagen) according to the manufacturer’s protocol. RNA concentration was measured with NanoDrop ND-1000 spectrophotometer (Thermo Fisher Scientific). Reverse transcription of RNA was performed using the MMLV RT kit (Evrogen) according to the manufacturer’s protocol. The obtained cDNA samples were stored at −20°C. qPCRmix-HS SYBR (Evrogen) was used for RT-PCR performed with DTprime detecting amplifier (DNA Technology).

The oligonucleotide primers used for RT-PCR were designed based on the mRNA sequences of the studied genes from the UCSC Genome Browser database (Kent et al., 2002). Primer selection was performed using Primer-BLAST software (Ye et al., 2012). The possibility of the formation of secondary structures (hairpins), homo- and heterodimers by the primers was assessed using OligoAnalyzer 3.1 software (Owczarzy et al., 2008). *EEF1A1* and *ACTB* were selected as reference genes (Maltseva et al., 2013). The sequences of the primers are presented in Supplementary Table S2. The evaluation of the differences in the expression of the selected genes was carried out using the software REST 2009 v.2.0.13 (Pfaffl et al., 2002; Vandesompele et al., 2002). For each group 3 independently obtained samples of RNA were used to assess expression levels of the selected genes.

### 2.4 Gene Ontology enrichment analysis

Analysis of the enriched biological processes among the genes with increased and decreased expression was carried out using Gene Ontology (GO) database (Ashburner et al., 2000; Gene and Consortium, 2019) and “topGO” package for R programming language. The results were obtained using “weight01″ algorithm, *p*-values were calculated using Fisher’s exact test.

### 2.5 Analysis of cellular fatty acids composition

To analyze the composition of intracellular fatty acids, the cells were cultured in culture flasks with surface area of 25 cm^2^ in 5 mL of complete nutrient medium for 48 h. Samples containing pellets of 1.5 × 10^6^ million cells were prepared for HPLC-MS analysis following a protocol based on a previously published method (Valianpour et al., 2003). The experiment was carried out in two biological replicates.

### 2.6 Analysis of the kinetics of PUFAs uptake

To study the kinetics of absorption of LС-PUFAs by the cells from the nutrient medium, 2 × 10^5^ cells were seeded into the wells of 24-well culture plates in 500 µL of complete cell culture medium. The plates were then incubated in a cell culture incubator at 37°C, 5% CO_2_ (Sanyo) for 24 h. After that, the nutrient medium was replaced with a medium containing 50 μM of one of the studied LC-PUFAs (linoleic acid: LA, α-linolenic acid: ALA, arachidonic acid: AA, and docosahexaenoic acid: DHA) and the plates were incubated in the cell culture incubator until the end of the experiment. Sampling of the nutrient medium for analysis of the concentration of LC-PUFAs was carried out after 2, 4, 9, 22 and 27 h The experiment was carried out in two biological replicates.

To analyze the content of free fatty acids in the selected samples, each of them was combined 1:1 with methanol, thoroughly mixed by vortexing, and incubated at room temperature for 5 min. Next, the samples were centrifuged at 13,000 g for 5 min and the supernatant was transferred into chromatographic vials for analysis.

The composition of fatty acids in the samples was analyzed by HPLC using an Infinity 1200 chromatograph (Agilent Technologies) with a UV detector. Separation of fatty acids was carried out on a ZORBAX Eclipse XDB-C18 reverse-phase chromatographic column (length 150 mm, inner diameter 4.6 mm, sorbent particle diameter 5 µm) (Agilent Technologies), in the gradient elution mode (Supplementary Table S3). Phase A was deionized water with 0.1% vol. formic acid and phase B was acetonitrile with 0.1% vol. formic acid. The flow rate of the mobile phase was constant and equal to 1.5 mL/min. Fatty acid chromatograms were recorded with the UV detector at 194 nm. Concentrations of the analyzed PUFAs in the samples were determined based on the external calibration curves (Supplememtary Figure S1).

### 2.7 GPx4 activity assay

GPx4 activity analysis was performed with the Colorimetric Glutathione Peroxidase Assay Kit (Abcam) according to the standard protocol. Cell suspensions of 2 × 10^6^ were used for each test. GPx4 activity was assessed by measurement of the decrease in NADPH absorbance at 340 nm with X-mark plate reader (BioRad). One unit is defined as the amount of enzyme that will cause the oxidation of 1.0 µmol of NADPH to NADP+ under the assay kit condition per minute at 25°C.

### 2.8 ROS accumulation assay

The cells were seeded into 96-well plates (20,000 cells/well) and incubated overnight. After 24 h, PUFAs in different concentrations were added into the wells and the plates were incubated for 4 h. Next, the culture medium was removed from the wells, the cells were washed with DPBS, and solution of the dye-indicator of reactive oxygen species H2DCFDA (cell-permeant 2′,7′-dichlorodihydrofluorescein diacetate, final concentration 10 µM) was added, and the plates were incubated for 30 min. After 30 min, the dye was removed, the wells were washed with DPBS and fresh DPBS was added into each well. Then integral fluorescence signal (excitation at 485 nm, emission at 535 nm) was measured on the SpectraMax i3 Plate Reader (Molecular Devices). Photomicrographs of cells were obtained using an inverted fluorescent microscope Axio Observer Z1 (Carl Zeiss).

### 2.9 Lipid droplets formation assay

MDA-MB-231 cells were seeded in a 96-well plate (15∙10^3^ cells per well), grown overnight and then cultured with 50 μM of various LC-PUFAs for 24 h.

For lipid droplets staining by Oil Red O, cells were washed twice with PBS, fixed with 10% formalin for 45 min, and then rinsed twice in water for 1 min, followed by 5 min in 60% isopropanol. The cells were stained with Oil Red O (1.8 mg/mL in 60% isopropanol) for 15 min and rinsed 5 times with ddH_2_O to remove excess stain. Oil Red O stained cells were directly visualized and imaged using an inverted fluorescent microscope Axio Observer Z1 (Carl Zeiss). Quantification of lipid accumulation was achieved by Oil Red O extraction with 100% isopropanol and gentle agitation for 5 min at room temperature. Then the extracts were transferred to a new 96-well plate and absorbance was measured at 492 nm using X-mark plate reader (BioRad).

For lipid droplet staining with BODIPY, cells were washed twice with DPBS, fixed with 10% formalin for 45 min and then washed twice with DPBS for 1 min. Cells were incubated with 2 μM BOBIPY (Lumiprobe) in the dark at 37°C for 60 min and then washed 3 times with DPBS to remove excess dye. Cells stained with BOBIPY were visualized directly with an inverted fluorescent microscope Axio Observer Z1 (Carl Zeiss).

### 2.10 Cell viability assay

The viability of tumor cells in all cases was determined using the CellTiter 96® AQueous One Solution (Promega) according to the manufacturer’s recommendations. Optical density was measured at 490 nm with X-mark plate reader (BioRad). All analyzed LC-PUFAs were dissolved in ethanol and all other small molecules were dissolved in DMSO.

For the experiments with 2D cultures, 1 × 10^4^ MDA-MB-231 cells per well were seeded into 96-well plates and incubated (37°C, 5% CO2) for 24 h with a tested compound or a combination of compounds. Then the viability was measured. Working concentrations of Erastin and Ferrostatin-1 were choosen based on previously published data (2–80 μM for Erastin and 0.1–2 μM for Ferrostatin-1) (Dixon et al., 2014; Yu et al., 2019; Anthonymuthu et al., 2021; Li et al., 2021; Chen et al., 2022; Wang et al., 2022).

To generate spheroids, MDA-MB-231 cells were cultured in Matrigel Growth Factor Reduced (GFR) Basement Membrane Matrix (Corning) in 24-well plates for 48 h. Then the spheroids were diluted in Matrigel Growth Factor Reduced (GFR) Basement Membrane Matrix (Corning) and seeded into 96-well plate. After solidification of the gel, complete cell culture medium was added into each well.

After 24 h, the cell culture medium was replaced with the control medium or the medium containing single standard of care (SOC) drugs. Clinically relevant concentrations were used in the assay: 371.7 μM for 5-FU, 5.47 μM for Docetaxel, 6.73 μM for Doxorubicin, 89.3 μM for Gemcitabine, 1.75 μM for Methotrexate, and 811 nM for Vinorelbine (Liston and Davis, 2017). Then the cells were incubated for 3 h in cell culture incubator (37°C, 5% CO2), and the medium was replaced with fresh complete cell culture medium or the medium containing Erastin (1 μM) or DHA (100 μM). Then plates were incubated in cell culture incubator (37°C, 5% CO2) for 72 h, and relative number of viable cells was measured. Growth rate of cancer cells was calculated as described previously (Hafner et al., 2016).

ANOVA with Tukey *post hoc* test has been used to assess statistical significance of the changes in viability or growth rate. The differences were considered statistically significant if *p*-values were less than 0.05.

### 2.11 Apoptosis assay

To study the activation of apoptosis Dead Cell Apoptosis Kit with Annexin V Alexa Fluor ™ 488 & Propidium Iodide (PI) (Thermo Fisher Scientific) was used according to the manufacturer’s instructions. Cells were treated with different PUFAs then detached and analyzed as described earlier (Nikulin et al., 2021). Analysis of raw data was carried out using FlowJo 10.6.1 software. Chi-squared test with Bonferroni correction for multiple comparisons were used to access statistical significance of the changes in different populations.

## 3 Results

### 3.1 Knockdown of *IGFBP6* gene leads to multiple changes in lipid metabolism

As one gene from our prognostic pair, *ELOVL5*, is directly involved in the elongation of fatty acids, we reanalyzed our previously published transcriptomic (GSE165854) and proteomic (PXD023892) data for a more detailed view of the changes in the molecular pathways related to lipid metabolism Surprisingly, we found a number of biological processes associated with lipid metabolism that were significantly changed not only after knockdown of *ELOVL5*, but of *IGFBP6* as well (Table 1; Table 2). In addition, a lot of the genes involved in these processes changed their expression by at least 1.5 times (FDR *p* < 0.05) after the knockdown of *IGFBP6* gene (Supplementary Figures S2–S16).

**TABLE 1:** Significantly changed (*p* < 0.05) Gene Ontology biological processes after knockdown of *IGFBP6* gene according to the transcriptomic analysis.

Up-regulated genes		Down-regulated genes	
GO ID	Description	GO ID	Description
GO:0046949	fatty-acyl-CoA biosynthetic process	GO:0046320	regulation of fatty acid oxidation
GO:0008654	phospholipid biosynthetic process	GO:0019216	regulation of lipid metabolic process
GO:0046479	glycosphingolipid catabolic process	GO:0046474	glycerophospholipid biosynthetic process
		GO:0010888	negative regulation of lipid storage
		GO:0045332	phospholipid translocation
		GO:0046839	phospholipid dephosphorylation

**TABLE 2:** Significantly changed (*p* < 0.05) Gene Ontology biological processes after knockdown of *IGFBP6* gene according to the proteomic analysis.

Up-regulated genes		Down-regulated genes	
GO ID	Description	GO ID	Description
GO:0008654	phospholipid biosynthetic process	GO:0006633	fatty acid biosynthetic process
		GO:0019217	regulation of fatty acid metabolic process
		GO:0071398	cellular response to fatty acid
		GO:0032365	intracellular lipid transport
		GO:0045017	glycerolipid biosynthetic process
		GO:0046890	regulation of lipid biosynthetic process
		GO:0019915	lipid storage

**FIGURE 1:**
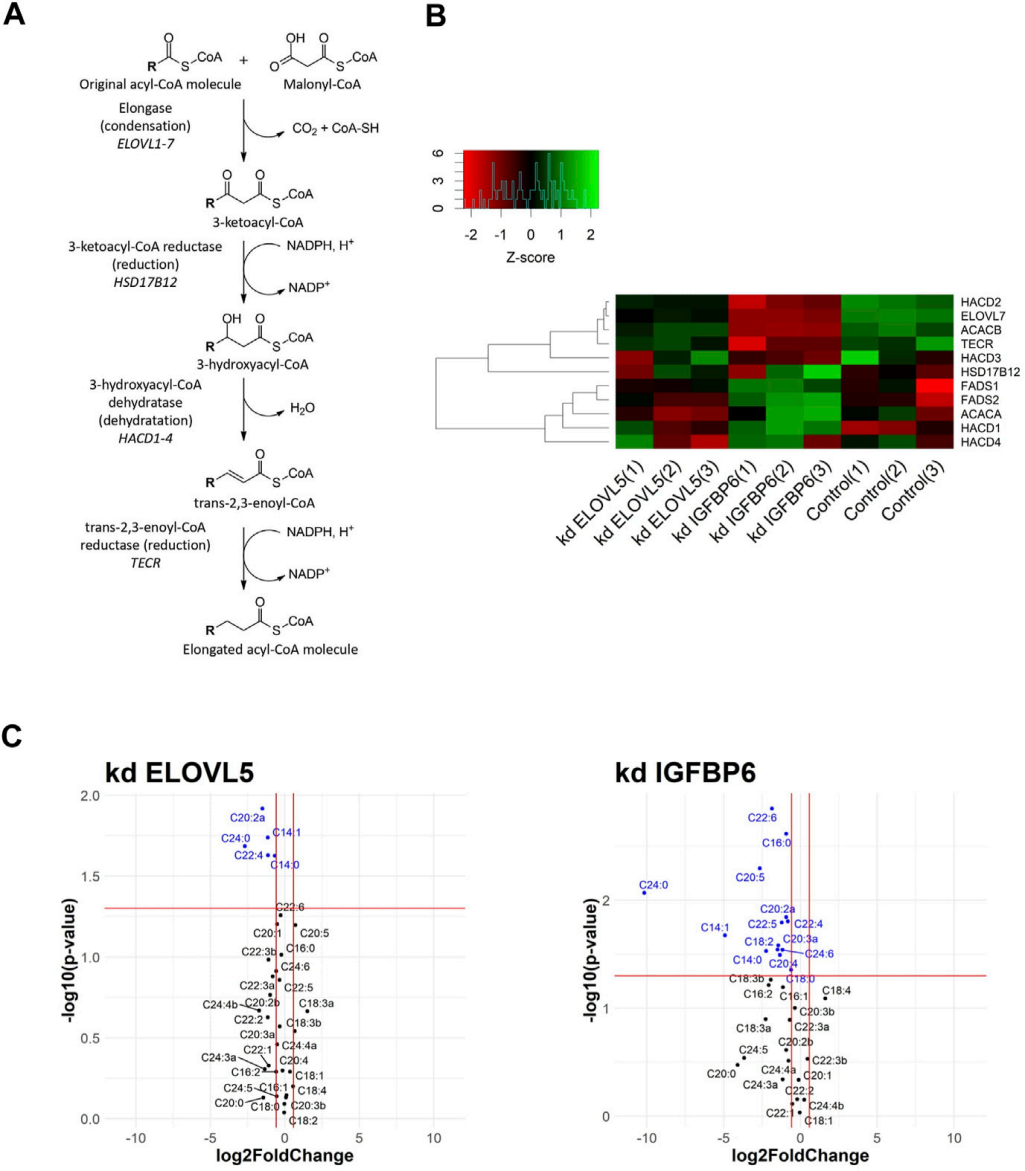
**(A)** Schematic drawing of fatty acid elongation. R represents a fatty acid with a varying length. **(B)** Heatmap of gene expression for selected genes from fatty acids elongation pathway. **(C)** Volcano plots showing fold changes and *p*-values of fatty acids content (kd *ELOVL5* vs. Control and kd *IGFBP6* vs. Control).

Fatty acid biosynthesis was one of the altered biological processes. Elongation is the key step of this pathway (Figure 1A). In the first stage, the condensation reaction of acyl-CoA with malonyl-CoA catalyzed by ELOVL1-7 enzymes occurs, followed by three consecutive reactions catalyzed by enzymes 3-ketoacyl-CoA reductase (*HSD17B12* gene), 3-hydroxyacyl-CoA dehydratase (*HACD1-4* genes), and trans-2,3-enoyl-CoA reductase (*TECR* gene) (Moon and Horton, 2003; Leonard et al., 2004; Ikeda et al., 2008). Malonyl-CoA, necessary for the elongation of long fatty acids, is synthesized by enzyme acetyl-CoA carboxylase (*ACACA* and *ACACB* genes).

According to the transcriptomic analysis (Figure 1B), expression of several genes from the fatty acids elongation pathway decreased as a result of the knockdown of *IGFBP6* gene in MDA-MB-231 cells; namely: *ACACB* (2.1 times, FDR *p* = 6.3·10^–9^), *HACD2* (2.1 times, FDR *p* = 1.6·10^–8^), and *TECR* (2.0 times, FDR *p* = 5.8·10^–6^). At the same time, the expression of the *HACD1* gene slightly increased (1.6 times, FDR *p* = 2.8·10^–3^). A decrease in the content of the TECR protein in MDA-MB-231 cells with the knockdown of *IGFBP6* gene was also confirmed by proteomic analysis (1.9 times, FDR *p* = 2.0·10^–2^).

Interestingly, according to the transcriptomic analysis the expression of one of the fatty acids elongases, *ELOVL7*, substantially dropped as a result of the knockdown of *IGFBP6* gene (4.2 times, FDR *p* = 2,4·10^–10^), and it was confirmed by RT-PCR (10.4 times, *p* = 2,6·10^–2^). Moreover, similar but less prominent effect was observed after knockdown of *ELOVL5* gene. In this case expression of *ELOVL7* decreased 1.8 times (FDR *p* = 6,0·10^–4^). *ELOVL7* catalyzes elongation of very long chain saturated as well as unsaturated fatty acids, including n-3 and n-6 PUFAs (Naganuma et al., 2011).

Expression of several enzymes from the pathway of elongation of n-3 and n-6 polyunsaturated fatty acids, which include *ELOVL5*, changed after the knockdown of *IGFBP6* gene (Leonard et al., 2000; Wang et al., 2008; Moon et al., 2009). A slight but statistically significant increase in the expression of *ELOVL5*, *FADS1,* and *FADS2* genes after the knockdown of *IGFBP6* has been noted (*ELOVL5* 1.4 times, FDR *p* = 2.0·10^–2^; *FADS1* 1.3 times, FDR *p* = 2.0·10^–2^ and *FADS2* 1.5 times, FDR *p* = 3.0·10^–4^). The increase in *FADS1* and *FADS2* mRNAs was confirmed by RT-PCR (*FADS1* 2.7 times, *p* = 3.5·10^–2^ and *FADS2* 2.0 times, *p* = 3.3·10^–2^), and the increase in FADS2 protein level in MDA-MB-231 with knockdown of *IGFBP6* gene was also confirmed by proteomic analysis (3.4 times, FDR *p* = 4.2·10^–3^).

All described changes suggest that *IGFBP6* can be involved in the regulation of lipid metabolism in breast cancer cells. To our knowledge, there are no previously published data on the role of *IGFBP6* in the lipid metabolism; therefore, we performed additional experiments to test this relation.

We found that knockdowns of both *ELOVL5* and *IGFBP6* genes led to a significant change in the content of individual long and very long fatty acids in MDA-MB-231 cells (Figure 1C; Supplementary Table S4). In particular, when the *ELOVL5* gene is knocked down, the content of fatty acids C22:4n-6, C20:2 (peak “a", n-6 or n-9) decreased. Also, when either of the genes was knocked down, there was a significant decrease in the content of very long saturated fatty acid C24:0 in the cells. This result is in good agreement with the results of transcriptomic analysis, which demonstrated a decrease in the expression of the *ELOVL7* gene in both stable cell lines. Interestingly, after the knockdown of the *IGFBP6* gene, a significant decrease in the content of n-3 eicosapentaenoic (EPA, C20:5n-3) and docosahexaenoic (DHA, C22:6n-3) acids in cells was also noted.

**FIGURE 2:**
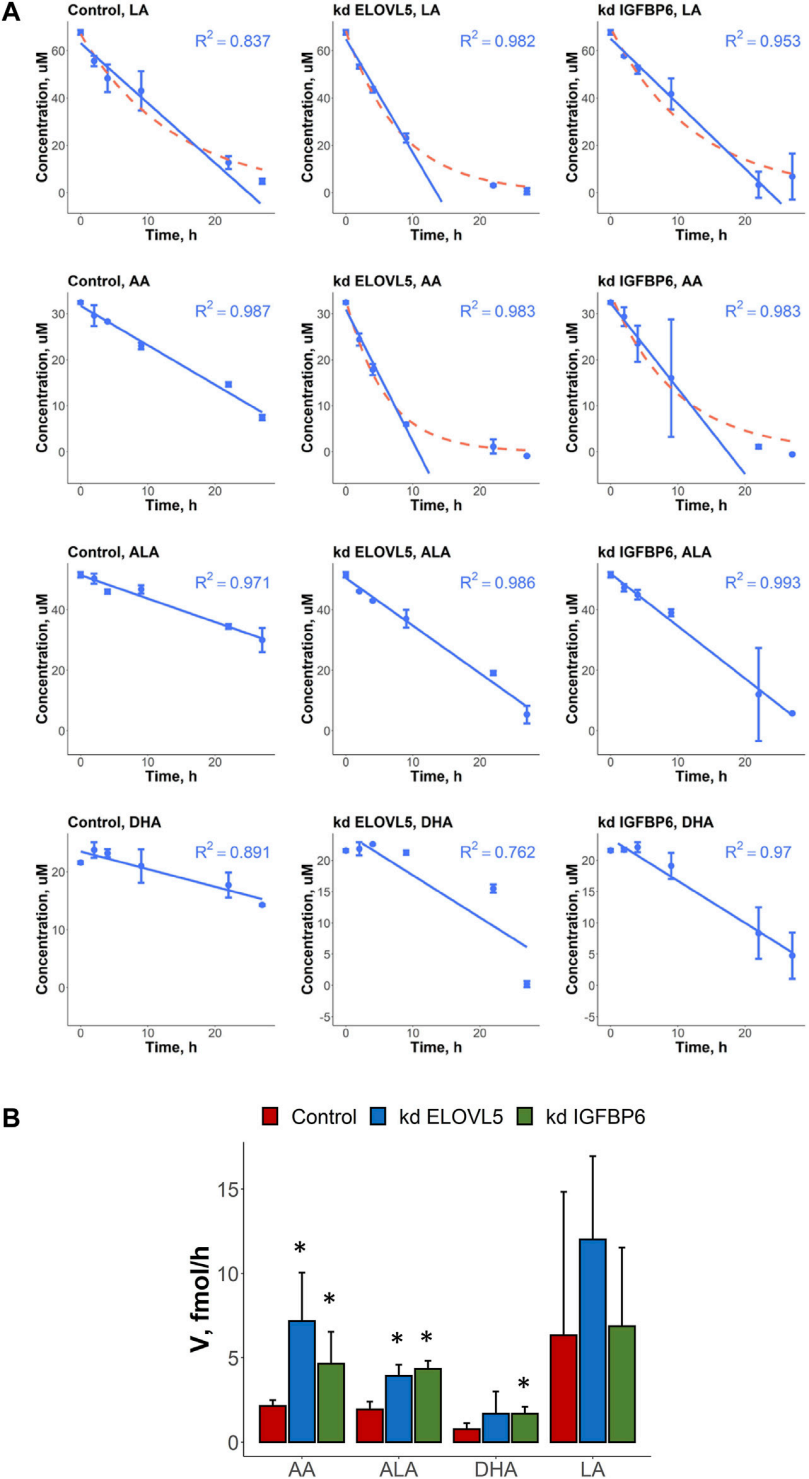
**(A)** Kinetics of changes in the concentration of various PUFAs (LA, AA, ALA and DHA) in cell culture medium in the presence of control MDA-MB-231 cells and the cells with knockdown of *ELOVL5* and *IGPBP6* genes. Error bars represent standard deviation (SD, *n* = 2). **(B)** Calculated uptake rates (per one cell) of various PUFAs (LA, AA, ALA and DHA) for control MDA-MB-231 cells and for the cells with knockdown of *ELOVL5* and *IGPBP6* genes. Error bars represent 95% confidence intervals. *—statistically significant difference versus control cells.

In this work we also measured uptake rates of four different PUFAs (LA, AA, ALA and DHA) by the control MDA-MB-231 cells and by the cells with knockdowns of *ELOVL5* and *IGFBP6* genes. In most cases, the kinetics of changes of PUFAs’ concentrations in cell culture medium could be accurately approximated by the linear model with constant uptake rate over the whole time range (Figure 2A; Supplementary Table S5). At the same time, an exponential model was a better alternative in several cases. Indicating a more efficient uptake, the cases following exponential kinetics evidently benefit from transport molecules and/or specific enzymes utilizing the fatty acid not becoming rate-limiting as the fatty acid concentration decreases. In particular, the kinetics of AA’s uptake quantitatively and qualitatively changed as a result of the knockdown of *ELOVL5* and *IGFBP6* genes. In both cases the cells with knockdowns demonstrated more efficient consumption.

To compare uptake rates of different PUFAs by different cells in cases of both linear and exponential kinetics, we approximated the initial parts of the exponential curves by straight lines and so obtained maximum initial uptake rates for those cases. We detected significant differences in the PUFAs’ uptake rates after the knockdowns of *ELOVL5* and *IGFBP6* genes (Figure 2B). For example, we found that uptake rate of AA increased 3.3 times as a result of the decrease in expression of *ELOVL5* gene. Similar, but less prominent change (2.2 times) was observed for *IGFBP6* gene. In addition, the consumption of ALA was 1.8 and 2.2 times faster after the knockdown of *ELOVL5* and *IGFBP6* genes respectively. And finally, uptake rate of DHA was significantly higher (2.2 times) for the cells with the knockdown of *IGFBP6* gene in comparison to the control cells. All other changes were statistically insignificant.

Interestingly, the expression of genes involved in the transport of fatty acids into the cell (Liu et al., 2015; Zhang et al., 2018) did not change significantly (FDR *p* > .05) after the knockdown of *ELOVL5* or *IGFBP6* gene (CD36, SLC27A1-6, FABP1-9 and FABP12 genes were analyzed). The observed changes in uptake rates are thus probably due to the changes in metabolism of LC-PUFAs.

### 3.2 Knockdowns of both *ELOVL5* and *IGFBP6* genes change the response of breast cancer cells to external fatty acids

**FIGURE 3:**
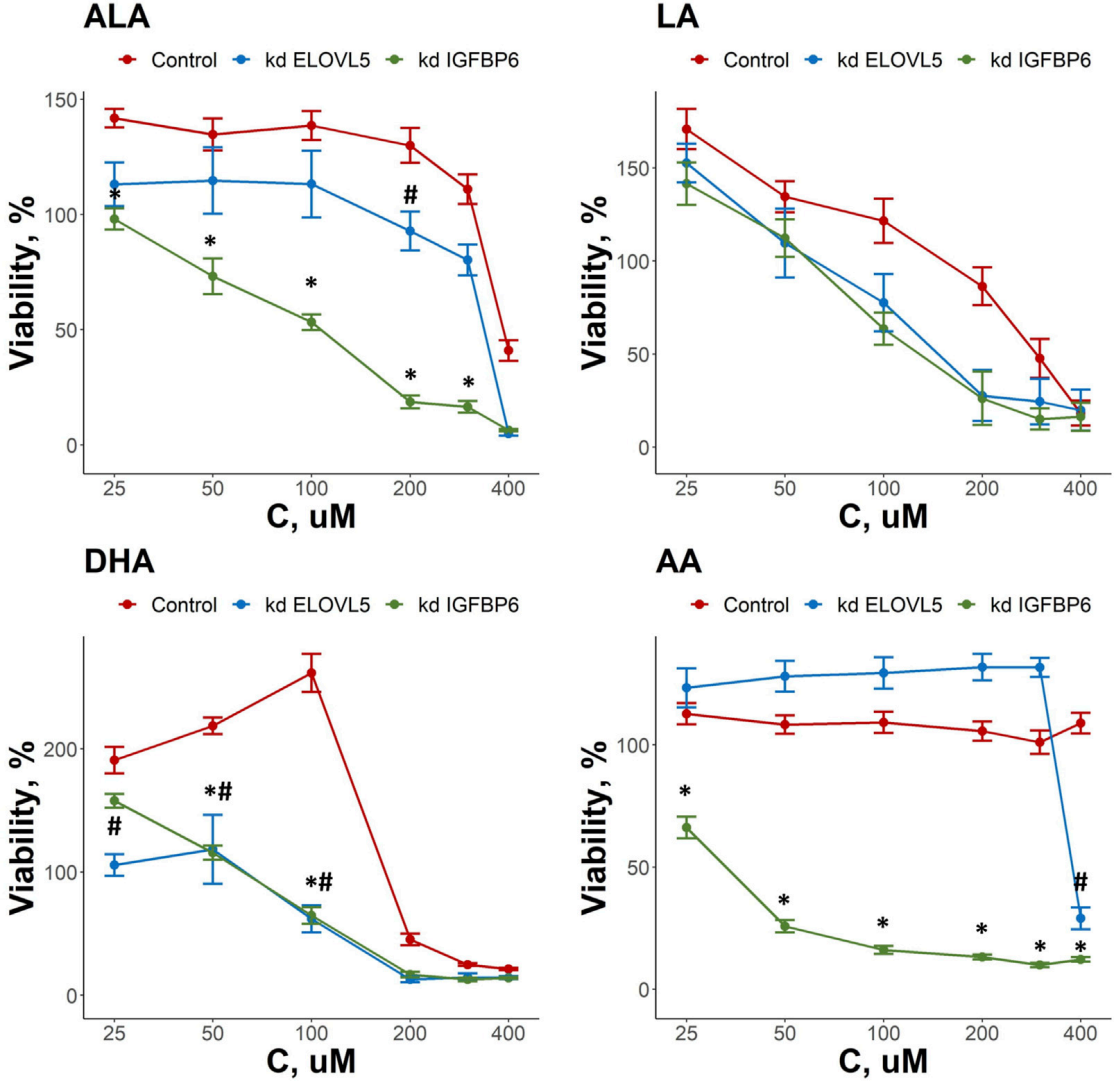
Effect of various LC-PUFA on the viability of control MDA-MB-231 cells and the cells with knockdown of *ELOVL5* and *IGFBP6* genes. Error bars represent standard error of mean (SEM, *n* = 3). *—*p* < 0.05 kd *IGPBP6* versus control cells. #—*p* < 0.05 kd *ELOVL5* versus control cells.

As both *ELOVL5* and *IGFBP6* genes affect lipid metabolism, we examined the changes in the response of MDA-MB-231 cells to external LC-PUFAs in the culture medium. Overall, we observed that all LC-PUFAs studied can decrease viability of breast cancer cells *in vitro* (Figure 3)*.* The viability of the cells with the knockdown of *IGFBP6* gene was significantly lower in comparison to the control cells for ALA in the range of 25–300 μM (*p* < 0.05), for DHA in the range of 50–100 μM (*p* < .05), and for AA in the range of 25–400 μM (*p* < .05). For the cells with reduced expression of *ELOVL5* gene, similar but less prominent effects were seen. The viability of these cells was lower than that of the control cells only at 200 μM for ALA (*p* < .05), at 400 μM for AA (*p* < 0.05), and in the range of 25–100 μM (*p* < .05) for DHA. The effect of LA was not significantly changed by either knockdown (*p* > .05). While n-3 ALA and n-6 AA turned out to be the least cytotoxic towards both control and knockdown *ELOVL5* cells, n-6 AA and n-3 DHA were highly cytotoxic to the knockdown *IGFBP6* cells, in line with the significantly greater sensitivity of the latter to all LC-PUFAs, and in good agreement with previous research (Schley et al., 2005). In all, our data demonstrate that reduction in the expression of *ELOVL5* or *IGFBP6* genes can lead to an increase in the sensitivity of breast cancer cells to various PUFAs.

Interestingly, we also found that some LC-PUFAs have a stimulating effect on the growth of MDA-MB-231 cells. For example, an increase in the viability of the control cells was observed in the range 25–200 μM for ALA, 25–100 μM for DHA and at 25 μM for LA. At the same time, the stimulating effect of these fatty acids on MDA-MB-231 cells with either *ELOVL5* or *IGFBP6* knockdown was less pronounced or completely absent.

### 3.3 Cytotoxic effect of PUFAs cannot be explained by apoptosis

**FIGURE 4:**
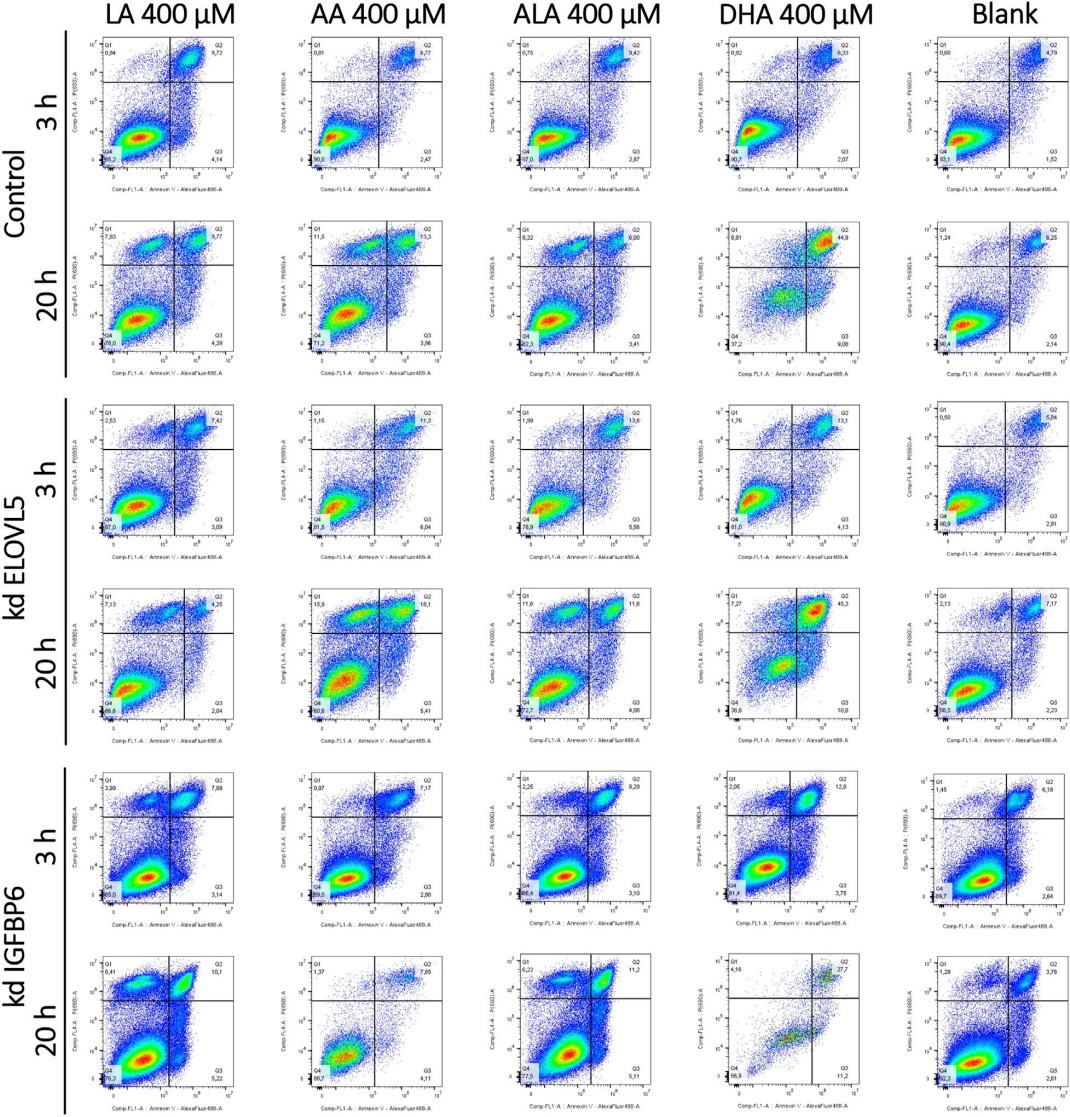
Effect of various LC-PUFAs on the activation of apoptosis in MDA-MB-231 cells. Two-dimensional plots of the integral fluorescence intensity of the annexin V conjugate with Alexa Fluor 488 dye (horizontal axis), and the integral fluorescence intensity of propidium iodide (vertical axis) in the cells with the knockdown of *ELOVL5* or *IGFBP6* genes, as well as in controls cells.

As all tested LC-PUFAs could decrease viability of the MDA-MB-231 breast cancer cells, we studied the effect of these LC-PUFAs on the activation of apoptosis in the cells. The analysis was carried out at 3 and 20 h after the addition of one of the LC-PUFAs (Figure 4; Supplementary Table S6). For the control MDA-MB-231 cells, 3 h after the addition of LA and ALA, a significant increase in the proportion of dead (AV + PI+) cells (from about 5% to 10%) was observed, accompanied by a decrease in the proportion of viable cells. When these fatty acids were added, the proportion of cells at the early stage of apoptosis also slightly increased from about 1.5% to 2.9%–4.1%. At 20 h after the addition of any LC-PUFA, there was a significant increase in the proportion of the control cells in the AV-PI+ region. Moreover, a pronounced peak with increased cell density was visualized in the two-dimensional density maps.

Objects in this area correspond to nuclear fragments without a cell membrane, which can result from necrosis (Sawai and Domae, 2011). Interestingly, during necrosis, cells can also first move into the AV + PI- region, mimicking the behavior of cells at an early stage of apoptosis, and only then move into the area of dead cells (AV + PI-) and into the area of nuclear fragments without a cell membrane. (AV-PI+) (Sawai and Domae, 2011). However, a significant increase in the proportion of cells in the AV-PI+ region is not characteristic of apoptosis, which makes it possible to distinguish between these two mechanisms of cell death (Wlodkowic et al., 2009; Sawai and Domae, 2011; Brauchle et al., 2015). An increase in the proportion of cells in the AV-PI+ is also characteristic of ferroptosis (Chen et al., 2017; Sun et al., 2018). At the same time the proportions of cells in the regions AV + PI+ and AV + PI- can also rise during ferroptosis (Chen et al., 2017; Sun et al., 2018).

The proportion of the control cells in the region corresponding to early apoptosis (AV + PI-) slightly increased 20 h after the addition of LA, ALA, and AA (from about 2% to 4%). The increase was more significant for the DHA (from about 2% to 9%). The proportion of dead cells also increased from 6% to 10%–13% for LA and AA, while the proportion of dead cells did not change for ALA. In case of DHA, most of the cells were in the AV + PI+ area of dead cells (about 45%), and not in the area corresponding to viable cells, as it was observed for other fatty acids.

For the knockdown *ELOVL5* cells, a significant increase in the proportion of dead cells and a decrease in the proportion of viable cells was observed 3 h after the addition of ALA, AA, and DHA. This effect was less pronounced for LA. After 20 h, the pattern for the knockdown *ELOVL5* cells was largely similar to the results observed in the control cells. One can notice a slightly increased sensitivity of the cells with the knockdown of *ELOVL5* gene to ALA and AA, which is in good agreement with the data of the MTS test.

Three hours after the addition of LA, the knockdown *IGFBP6* cells started to concentrate in the AV-PI+ region. Also, the number of dead knockdown *IGFBP6* cells became more pronounced when DHA was added, compared to the control cells. Other changes after 3 h were similar to the control cells. After 20 h, for shorter LA and ALA, clusters of the knockdown *IGFBP6* cells were observed in the AV-PI+ area. The proportions were similar to the control cells. For longer AA and DHA, much fewer events were observed and a significant increase of the proportion of the cells in the region of dead cells (AV + PI+) was also noticed. On the other hand, the increase of cell counts in the AV-PI+ area was less prominent.

Overall, the patterns of death observed here for all tested LC-PUFAs and the cell lines correspond more closely to necrosis or ferroptosis rather than apoptosis.

### 3.4 Knockdown of either *ELOVL5* or *IGFBP6* gene increases sensitivity to ferroptosis

**FIGURE 5:**
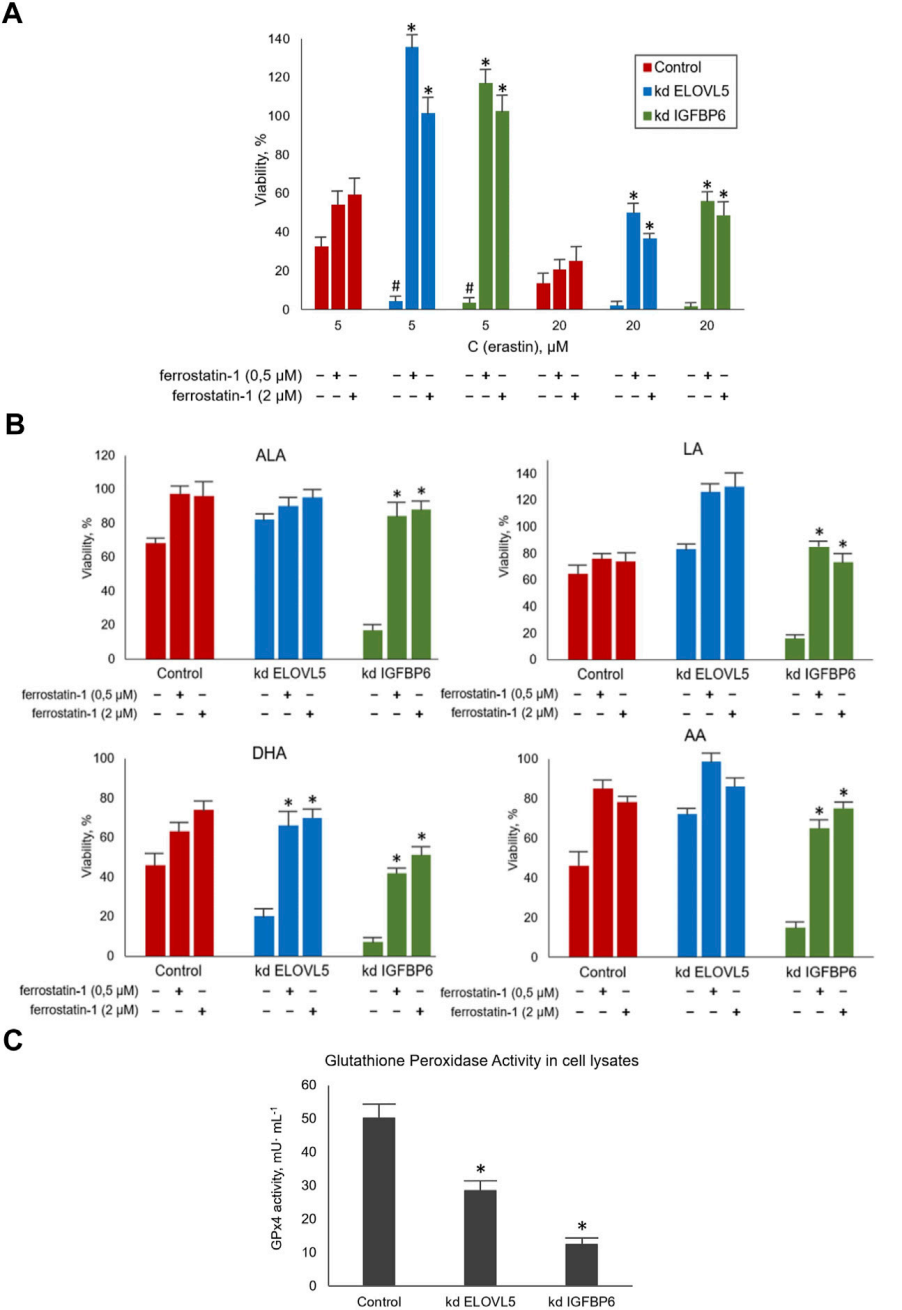
**(A)** Evaluation of the sensitivity of MDA-MB-231 cells to ferroptosis during their treatment with standard agents Erastin and Ferrostatin-1 in various combinations for 24 h. Error bars represent standard error of mean (SEM, *n* = 3). *—*p* < 0.05 versus pure Erastin. #—*p* < 0.05 versus control cells. **(B)** Evaluation of the sensitivity of MDA-MB-231 cells to ferroptosis during their treatment with LC-PUFA and Ferrostatin-1 for 24 h. The concentration for LA, AA, and ALA was 300 μM and for DHA 200 μM. Error bars represent standard error of mean (SEM, *n* = 3). *—*p* < 0.05 versus pure PUFA. **(C)** Glutathione Peroxidase Activity measured in cell lysates per one million cells after 10 min of incubation. Error bars represent standard error of mean (SEM, *n* = 3). *—*p* < 0.05 versus control cells.

To assess how the sensitivity of MDA-MB-231 cells to ferroptosis changes upon the knockdown of *ELOVL5* or *IGFBP6* genes, we treated the cells with ferroptosis inducer Erastin. A ferroptosis inhibitor Ferrostatin-1 was used to prevent the action of Erastin. Our data showed (Figure 5A) that the knockdown of both genes leads to a significant increase in sensitivity to ferroptosis of MDA-MB-231 cells (*p* < 0.05). Moreover, the effect of Erastin was significantly reduced when Ferrostatin-1 was added to both knockdown cells (*p* < 0.05), while the increase of viability was insignificant for the control cells (*p* > 0.05). Thus, the reduction in the expression of *ELOVL5* or *IGFBP6* genes leads to increased sensitivity to the induction of ferroptosis in breast cancer cells and at the same time makes them more responsive to its inhibition.

Next, we treated MDA-MB-231 cells with combinations of various LC-PUFAs and Ferrostatin-1. Ferrostatin concentrations were the same as in the previous experiment. The choice of working concentrations of fatty acids (300 μM for LA, AA, and ALA, and 200 μM for DHA) was made on the basis of the data obtained on the cellular response to external LC-PUFAs to ensure the cells showed a measurable decrease in viability.

We confirmed that increasing the content of LC-PUFAs in the nutrient medium decreases the viability of MDA-MB-231 cells (Figure 5B). This effect was significantly reduced by Ferrostatin-1 in the knockdown *IGFBP6* cells for all LC-PUFAs (*p* < 0.05) and in the knockdown *ELOVL5* for DHA (*p* < 0.05). Again, the action of Ferrostatin-1 was insignificant for the control cells (*p* > 0.05). Overall, these data suggest that the mechanisms of cell death induced by Erastin and LC-PUFAs are similar.

GPx4 peroxidase is one of the key enzymes involved in the glutathione pathway of ferroptosis inhibition. It oxidizes glutathione to form glutathione disulfide (GSSG) and reduces the cytotoxic lipid peroxides L-OOH to alcohols simultaneously. Disruption of GPx4 activity leads to a decrease in antioxidant capacity and, consequently, to ferroptosis. Our transcriptomic analysis revealed a significantly reduced level of GPx4 expression in knockdown *IGFBP6* cells compared to the control cells.

Therefore, the next important task was to check the activity of this enzyme in all three cell lines. In this assay, GSSG formed by GPx4 is reduced back to 2GSH by glutathione disulfide reductase (GR) using NADPH as a source of electrons and cumene hydroperoxide as a substrate. The rate of oxidation of NADPH is directly reflects the activity of GPx4. This rate was determined by following the change in absorbance of NADPH at 340 nm with a spectrophotometer in kinetic mode and all reactants kept in excess so as to make the action of GPx4 rate limiting in the oxidation of NADPH.

The results obtained (Figure 5C) demonstrated a significant decrease in GPx4 activity in knockdown *IGFBP6* cells compared to the control (*p* < 0.05). These data are in good agreement with the transcriptomic analysis. It should also be noted that the activity of this enzyme also slightly changed after the knockdown of *ELOVL5* gene (*p* < 0.05). Thus, the decrease in GPx4 activity is likely to be the key to lowering antioxidant capacity and increasing cells’ sensitivity to ferroptosis with the knockdowns.

Next, we tested whether the expression of *GPX4* gene can also decrease simultaneously with the expression of *IGFBP6* gene in breast cancer tissue. The analysis of the publicly available databases of transcriptomes of breast cancer samples showed that *GPX4* gene expression positively correlates with *IGFBP6* gene expression (i.e. decreases with a decrease in *IGFBP6* gene expression) in tumor samples from patients with ER+ breast cancer in 4 analyzed data sets (in total 10 datasets of ER+ breast cancer were analyzed) and in 5 datasets of ER-breast cancer patients (in total 7 datasets of ER-breast cancer were analyzed). Negative correlations have not been observed in this study.

To check whether the knockdown of either *ELOVL5* or *IGFBP6* gene leads greater accumulation of reactive oxygen species (ROS) in the cells upon addition of external PUFAs, we used ROS indicator 2′,7′-dichlorodihydrofluorescein diacetate (DCFDA, also known as H2DCFDA, DCFH-DA, and DCFH). DCFDA is a cell-permeable chemically reduced form of fluorescein. After cleavage of the acetate groups by intracellular esterases and oxidation, the non-fluorescent form of DCFDA is converted to highly fluorescent DCF.

**FIGURE 6:**
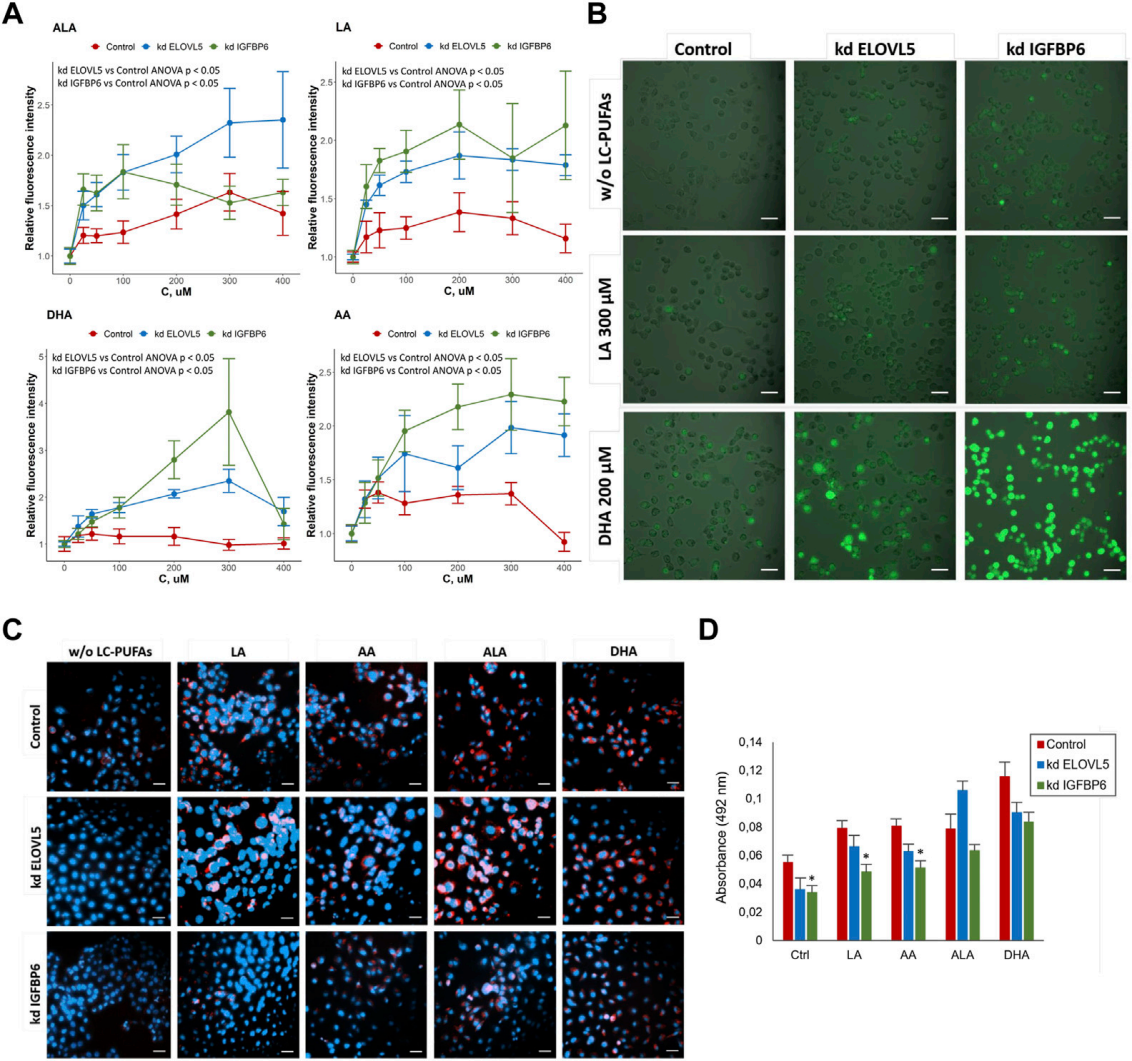
**(A)** Assessment of oxidative stress in MDA-MB-231 cells under the influence of various LC-PUFA by detection of reactive oxygen species (ROS) with H2DCFDA. Error bars represent standard error of mean (SEM, *n* = 3). **(B)** Photo of MDA-MB -231 cells in the cell culture medium without external LC-PUFAs and with addition of 300 μM LA or 200 μM DHA (staining with H2DCFDA). Scale bars indicate 50 μm. **(C)** Lipid droplets staining in MDA-MB-231 cells in control medium and in the medium containing 50 μM of LA, AA, ALA and DHA. Scale bars indicate 50 μm. Nuclei (blue) were stained with 4′,6-diamidino-2-phenylindole (DAPI), lipid droplets (red) were stained with Oil Red O. **(D)** Quantification of lipid droplets in MDA-MB-231 cells after treatment with various LC-PUFAs. The diagram represents absorbance of extracted Oil Red O. Error bars represent standard error of mean (SEM, *n* = 3).

The results showed (Figure 6A) that for all tested PUFAs fluorescence intensity of DCF normalized to the control culture medium without external PUFAs was higher for the cells with reduced expression of *ELOVL5* (ANOVA *p* < 0.05) and *IGFBP6* gene (ANOVA *p* < 0.05), indicating greater accumulation of ROS in the knockdowns. In addition, knockdown *IGFBP6* cells with almost all fatty acids, and especially DHA, demonstrated the highest increase in fluorescence intensity. These data have been confirmed by microscopic observations (Figure 6B). Overall, the greater accumulation of ROS in the knockdowns is in good agreement with lower activity of GPx4 and higher sensitivity of these cells to PUFAs.

One possible way to prevent oxidation of PUFAs is to store them in lipid droplets (Wang et al., 2017; Dierge et al., 2021). We compared the accumulation of lipid droplets in the cells with knockdowns to the control cells with the help of lipid dye Oil Red O. The results (Figure 6C) showed an increase in the accumulation of lipids in MDA-MB-231 cells after addition of external PUFAs to the nutrient medium. Oil Red O extraction was carried out for quantification of lipid accumulation (Figure 6D). We found that knockdown of *IGFBP6* gene significantly reduces the formation of lipid droplets under the control conditions (*p* <0.05) and after treatment with LA (*p* < 0.05) and AA (*p* < 0.05). These data were confirmed by staining the cells with another lipid dye BODIPY (Figure S17).

These results are in good agreement with the transcriptomic analysis, which demonstrated significantly reduced expression levels of the *AGPAT3*, *GPAT2*, *GPAT3*, and *DGAT1* enzyme genes in the knockdown *IGFBP6* cells compared to the control cells (Supplementary Table S7). These enzymes play important roles in the regulation of triacylglycerol (TAG) biosynthesis: *AGPAT3* and *GPAT2* translocate TAG molecules from the endoplasmic reticulum (ER) into lipid droplets (LD) during membrane fusion; *GPAT3* catalyzes the conversion of glycerol-3-phosphate to lysophosphatidic acid during the synthesis of triacylglycerol; and *DGAT1* catalyzes the conversion of diacylglycerol and fatty acid esters of coenzyme A to triacylglycerols (Wilfling et al., 2014; Wang et al., 2017; Quiroga et al., 2021). Thus, disruption of TAG biosynthesis and lipid droplet formation in the knockdown *IGFBP6* cells appear to be one of the reasons for their increased sensitivity to external LC-PUFAs.

### 3.5 Combination of SOC drugs with inducers of ferroptosis

To find out whether induction of ferroptosis by a PUFA or Erastin can significantly improve cytotoxic effects of standard of care (SOC) chemotherapeutic drugs, we tested different combinations on 3D cell models from control MDA-MB-231 cells, as well as from the cells with the knockdowns. The cells were grown in Matrigel and formed spheroids. This method allows one to create more physiologically relevant environment and provides a more gradual access for the drugs to the cells, reflecting better the conditions *in vivo*.

**FIGURE 7:**
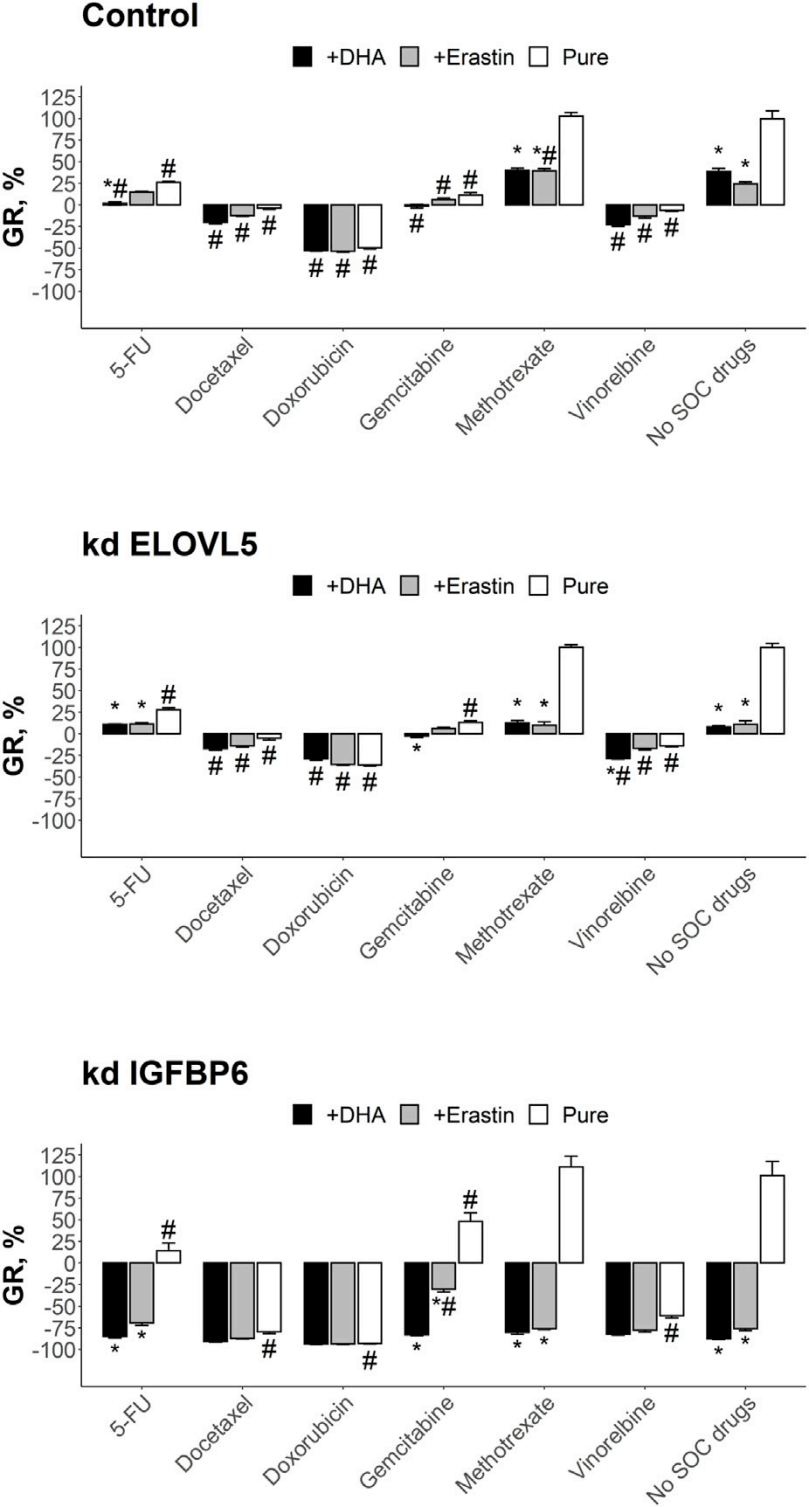
Results of the drug test for the tested SOC drugs and their combination with DHA and Erastin on the spheroids in Matrigel from MDA-MB-231 cells. Error bars represent standard error of mean (SEM, *n* = 3). *—*p* < 0.05 versus corresponding pure SOC treatment (without DHA or Erastin). #—*p* < 0.05 versus corresponding condition without SOC drugs (no SOC drugs).

Overall, almost all tested drugs at clinically relevant concentrations were able to significantly reduce growth rate or even cause cell death (Figure 7). The only exception was methotrexate which was inactive on all the three cell types (*p* > 0.05). As expected, breast cancer cells after the knockdown of either *ELOVL5* or *IGFBP6* gene were more sensitive to DHA (*p* < 0.05). Interestingly, after the knockdown of *IGFBP6* gene cells also became more sensitive to docetaxel, doxorubicin, vinorelbine and pure erastin (*p* < 0.05). On the other hand, decrease in the expression of *IGFBP6* gene led to higher resistance to gemcitabine (*p* < 0.05).

Analysis of the results of the combined treatment showed that combination of 5-FU with DHA resulted in better growth inhibition than pure 5-FU (*p* < 0.05) and pure DHA (*p* < 0.05) for control cells. The effect of this combination was still better than pure 5-FU for the cells with knockdown of *ELOVL5* (*p* < 0.05) or *IGFBP6* (*p* < 0.05) gene, however it was indistinguishable from pure DHA (*p* > 0.05), as DHA itself was much more potent for these cells. The effect of the combination of 5-FU with erastin on the control cells was almost the same as the effects of pure 5-FU (*p* > 0.05) and pure erastin (*p* > 0.05). However, erastin significantly improved the effect of 5-FU after the knockdown of *ELOVL5* (*p* < 0.05) or *IGFBP6* (*p* < 0.05) gene. But again, the result of the combination was indistinguishable from pure erastin (*p* > 0.05).

The inhibitory effect on growth of docetaxel in combination with either DHA or erasin was the same as the effect of pure docetaxel for all three cell types (*p* > .05). The same results were obtained for doxorubicin (*p* > 0.05). Very similar to these two drugs was vinorelbine with the exception for the cells with reduced expression of *ELOVL5* gene where the combination of vinorelbine with DHA resulted in slightly better growth inhibition than pure vinorelbine (*p* < .05) and pure DHA (*p* < 0.05). The effect of methotrexate in combination with DHA or erastin was in all cases better (*p* < .05) than the effect of pure methotrexate as it was not active as single drug. However, almost in all cases (with one exception for combination with erastin on the control cells) the effect did not differ from pure DHA (*p* > 0.05) or pure erastin (*p* > 0.05).

The results of the test for gemcitabine in combination with either DHA or erasin did not differ from pure gemcitabine on the control cells (*p* > 0.05). On the other hand, the combined treatment of gemcitabine with DHA outperformed pure gemcitabine after the knockdown of either *ELOVL5* (*p* < 0.05) or *IGFBP6* (*p* < 0.05) gene. However, as DHA was much more effective on these cells, the growth inhibitory effect of the combination was indistinguishable from pure DHA (*p* > .05). Addition of erastin to gemcitabine increased the effect of the later only on the cells with reduced expression of *IGFBP6* gene (*p* < 0.05).

Overall, these results clearly show that both DHA and erastin have the potential to inhibit growth of breast cancer cells. Moreover, in some cases they can enhance the effect of the SOC drugs, especially if the cells have a decreased expression of *IGFBP6* gene.

## 4 Discussion

In our previous work, we identified new transcriptomic prognostic markers of breast cancer relapse (Galatenko et al., 2015). According to those results, low expression of *ELOVL5* and *IGFBP6* genes corresponded to poor prognosis, however, their role in metastasis was unknown. Next, we examined properties of breast cancer cells related to metastatic propensity after the knockdown of either *ELOVL5* or *IGFBP6* gene (Nikulin et al., 2021). We found sharp increase in the expression of metalloproteinases and a decrease in cell adhesion, both of which are likely to promote invasion of cancer cells. We also carried out a pathway analysis and found that the knockdown of *IGFBP6* gene led to downregulation of biosynthesis of some fatty acids in which *ELOVL5* participates directly.

*ELOVL5* elongates n-3 and n-6 polyunsaturated fatty acids. It is known that its expression in breast cancer cells is elevated and that it directly affects the lipid composition in serum (Tomida et al., 2021). Moreover, the complete knockout of *ELOVL5* in the mouse breast carcinoma model led to a delay in tumor development and a decrease in tumor growth (Kieu et al., 2021). At the same time, the data on the impact of the *IGFBP6* gene on the lipid metabolism are limited. In this work we focused on the changes in lipid metabolism in breast cancer cells after both knockdowns, paying special attention to *IGFBP6*. Reanalysis of transcriptomic and proteomic data showed that the knockdown of *IGFBP6* gene significantly disrupted lipid metabolism at the mRNA and protein levels. Moreover, we detected significant changes in the composition of fatty acids in the cells after the knockdown of *IGFBP6* gene. Thus, our results suggest that *IGFBP6* can play an important role in the regulation of lipid metabolism in breast cancer cells.

Since blood plasma normally contains significant amount of fatty acids (Abdelmagid et al., 2015) and tumor cells often increase their consumption of fatty acids from the external environment as a result of metabolic reprogramming (Munir et al., 2019), we examined the rates of consumption of external LC-PUFAs and viability of MDA-MB-231 breast cancer cells in the presence of LC-PUFAs. We found significant increase in the uptake rates of several LC-PUFAs after the knockdown of either *ELOVL5* or *IGFBP6* gene. We also found that the effect of the LC-PUFAs is concentration dependent. At low concentration both n-3 and n-6 LC-PUFAs can stimulate growth of MDA-MB-231 breast cancer cells, but as the concentration rises, they become toxic for them. Interestingly, the toxicity was higher for the knockdown cells.

Overall, our results are in good agreement with previous data. n-3 LС-PUFAs have been previously shown to inhibit the proliferation, migration, and invasion of tumor cells *in vitro* (Chamras et al., 2002; Huang et al., 2017; Davison et al., 2018). On the other hand, n-6 LC-PUFAs can also induce cell death by increasing the content of reactive oxygen species (Zhang et al., 2015), but their inhibitory effects on tumor cells are either less pronounced compared to n-3 LC-PUFAs, or absent, or, on the contrary, stimulating effects are observed (Connolly and Rose, 1993; Gonzalez-Reyes et al., 2017). To date, there are also studies in the literature showing that subjects with a higher dietary n-3/n-6 ratio of LC-PUFAs have a significantly lower risk of breast cancer among the study populations (Zheng et al., 2013; Yang et al., 2014). However a recent meta-analysis found no effect of elevated n-3 LC-PUFAs levels on the risk of being diagnosed with any cancer (high-quality evidence) and likely on the risk of dying from cancer (Hanson et al., 2020). The key to this discrepancy can be the fact that the effect of LC-PUFAs is concentration dependent and the sensitivity of cancer cells to the action of LC-PUFAs depends on the transcriptional programs, in particular on the expression of the gene pair studied in this work.

To identify the cause of cell death caused by LC-PUFAs, we studied activation of apoptosis in the cells by flow cytometry. Overall, we observed very limited transition through the region of early apoptosis and a lot of cells in the region characteristic for other types of cell death. These results were notable as they indicated a non-apoptotic mode of cell death after exposure to external LC-PUFAs. Based on the previously published data, we considered that LC-PUFAs cause ferroptosis in breast cancer cells (Chen et al., 2021a; Dierge et al., 2021).

Ferroptosis is one of the mechanisms of programmed cell death, which is based on disturbance of the redox balance (Stockwell et al., 2017). Ferroptosis is fundamentally different from other programmed cell death mechanisms such as apoptosis, pyroptosis, and necroptosis. The term “ferroptosis” was introduced in 2012 to identify an iron-dependent mechanism of cell death with excessive lipid peroxidation followed by destruction of cell membrane. Since ferroptosis was discovered relatively recently, a number of aspects of its mechanism remain unknown to date. In particular, the effectors of this process have not yet been identified, although many of the signaling and metabolic cascades associated with ferroptosis are already known.

The molecular mechanism of ferroptosis is based on the regulation of the balance between oxidative damage and antioxidant protection (Chen et al., 2021b). The key stage in ferroptosis is elevated peroxidation of polyunsaturated fatty acids in phospholipids. There are several ways by which this process can be activated. Firstly, increased activity of the enzymes involved in the synthesis of polyunsaturated fatty acid esters, in particular ACSL4 and LPCAT3, can lead to an increase in peroxidation, since this increases the amount of available substrate for the reaction. Secondly, the activation of the enzymes that directly catalyze the oxidation reaction, for example, various lipoxygenases, can lead to the induction of ferroptosis. And finally, an increase in the concentration of reactive oxygen species due to the activation of proteins involved in their production, or by increasing the concentration of iron, also enhances peroxidation of polyunsaturated fatty acids. In addition, various antioxidant systems are involved in cell defense, in particular, the cascade of synthesis and utilization of glutathione (one of the key enzymes is glutathione peroxidase GPX4), as well as coenzyme Q. A decrease in the activity of these systems can also trigger ferroptosis.

Our data suggest that LC-PUFAs can trigger ferroptosis in breast cancer cells. Firstly, the results of the flow cytometry experiments were similar to the data published previously in the study of ferroptosis by this method (Chen et al., 2017; Sun et al., 2018). Secondly, our hypothesis was also supported by the experiments on the inhibition of the cell death caused by LC-PUFAs with canonical inhibitor of ferroptosis Ferrostatin-1, which can significantly increase cell viability in some cases. Moreover, we have found that the knockdown of either *ELOVL5* or *IGFBP6* gene significantly increases sensitivity of MDA-MB-231 cells to canonical inducer of ferroptosis Erastin, and again the cells can be rescued by Ferrostatin-1. Interestingly, previously it has been demonstrated that expression of *ELOVL5* gene can affect sensitivity to ferroptosis in gastric cancer (Lee et al., 2020).

The reason why the breast cancer cells with reduced expression of either *ELOVL5* or *IGFBP6* gene are more sensitive to ferroptosis is complex. First of all, we found that the cells with the knockdowns accumulate more reactive oxygen species in response to external LC-PUFAs. Partly this can be explained by higher uptake of these LC-PUFAs from external medium. However, we also found that a significant decrease in the activity of one of the main antioxidant enzymes, GPX4, resulted from the knockdowns. Moreover, our data suggest that the cells with knockdown of *IGFBP6* gene are less efficient in storage of fatty acids in lipid droplets, and thus more substrate for peroxidation can be available in these cells. In addition, there is evidence in the literature that an increase in *IGFBP6* may improve mitochondrial fitness and redox status based on a decrease in mitochondrial ROS production (Longhitano et al., 2022).

At present, it is known that tumor cells are often resistant to classical mechanisms of programmed cell death, such as apoptosis. At the same time, ferroptosis is considered as a promising alternative means to destroy the tumor (Wu et al., 2020; Chen et al., 2021b). The accumulated information indicates that therapy-resistant tumor cells (in particular, the cells that have undergone epithelial to mesenchymal transformation or tumor stem cells) are sensitive to the induction of ferroptosis (Viswanathan et al., 2017; Cosialls et al., 2021). The influence of the expression of the genes associated with ferroptosis on the prognosis of some tumors (in particular, prostate cancer and colorectal cancer) has also been established (Wang Y. et al., 2021; Lv et al., 2021). Based on these data, ferroptosis inducers are considered to be a promising new class of anticancer drugs (Wang H. et al., 2021). Moreover, it has been proven that some of the approved drugs also trigger ferroptosis in tumor cells (Su et al., 2020).

Despite the fact that the effect of n-3 LC-PUFAs on non-tumorogenic cells is not fully understood, some studies showed that at concentrations which inhibit the growth of tumor cells, n-3 LC-PUFAs have little or no cytotoxic effect on normal breast cells (Grammatikos et al., 1994; Bernard-Gallon et al., 2002). Thus, as induction of ferroptosis is considered to be a promising means to kill cancer cells and potential harm of LC-PUFAs to normal cells is low, we studied combinations of the standard of care (SOC) chemotherapeutic drugs with DHA (an n-3 LC-PUFA). Our results showed that in some cases induction of ferroptosis can enhance the effect of the SOC drugs, especially if the cells have decreased expression of *IGFBP6* gene. Thus, it would be worthwhile to test LC-PUFAs in combination with anti-cancer drugs in more clinically relevant settings.

## 5 Conclusion

In this work we showed that, to our surprise, the knockdown of *IGFBP6* gene, a member of the IGF-binding protein family, led to significant changes in lipid metabolism in MDA-MB-231 cells. It was also found that a decrease in the expression of either *IGFBP6* or *ELOVL5* gene increases sensitivity of MDA-MB-231 breast cancer cells to LC-PUFAs and our data suggest that they cause cell death by activation of ferroptosis. We suspect a significant drop in the activity of the main antioxidant enzyme GPX4 in the cells after the knockdowns is the key reason for this phenomenon. Moreover, observed changes in the lipid metabolism after the knockdown of *IGFBP6* gene and increased uptake of some PUFAs can also contribute to it. Use of standard chemotherapeutics in combination with ferroptosis inducers showed that in some cases the latter can significantly enhance the effect of the drugs, especially for the cells with low expression of *IGFBP6* gene. Thus, for the breast cancer patients with low expression of *IGFBP6* and *ELOVL5* genes in cancer tissue the addition of PUFAs to the chemotherapy regimen can be potentially beneficial and should be tested in more clinically relevant settings.

## Data Availability

Publicly available datasets were analyzed in this study. This data can be found here: https://www.ncbi.nlm.nih.gov/geo/query/acc.cgi?acc&equals;GSE165854 https://www.ebi.ac.uk/pride/archive/projects/PXD023892.
