# Supplementary material for: ELOVL5 and IGFBP6 genes modulate sensitivity of breast cancer cells to ferroptosis
Source: Front Mol Biosci. 2023 Jan 13;10:1075704. doi: 10.3389/fmolb.2023.1075704 (PMC9880435; doi:10.3389/fmolb.2023.1075704)
Supplement: Supplementary file 1 [file DataSheet1.PDF]

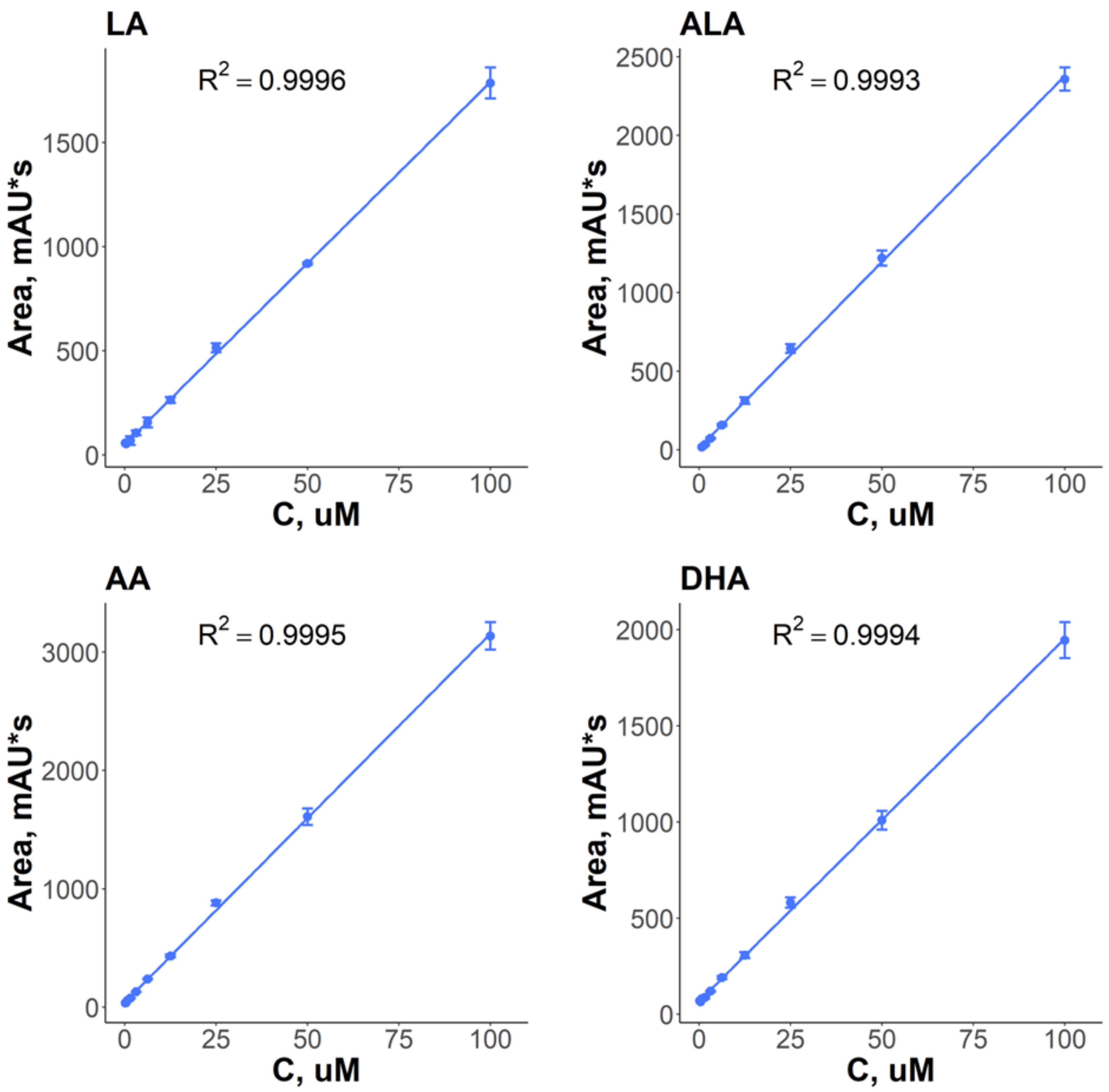

Figure S1. Calibration curves for various PUFAs (LA, AA, ALA and DHA). Error bars represent standard deviation.

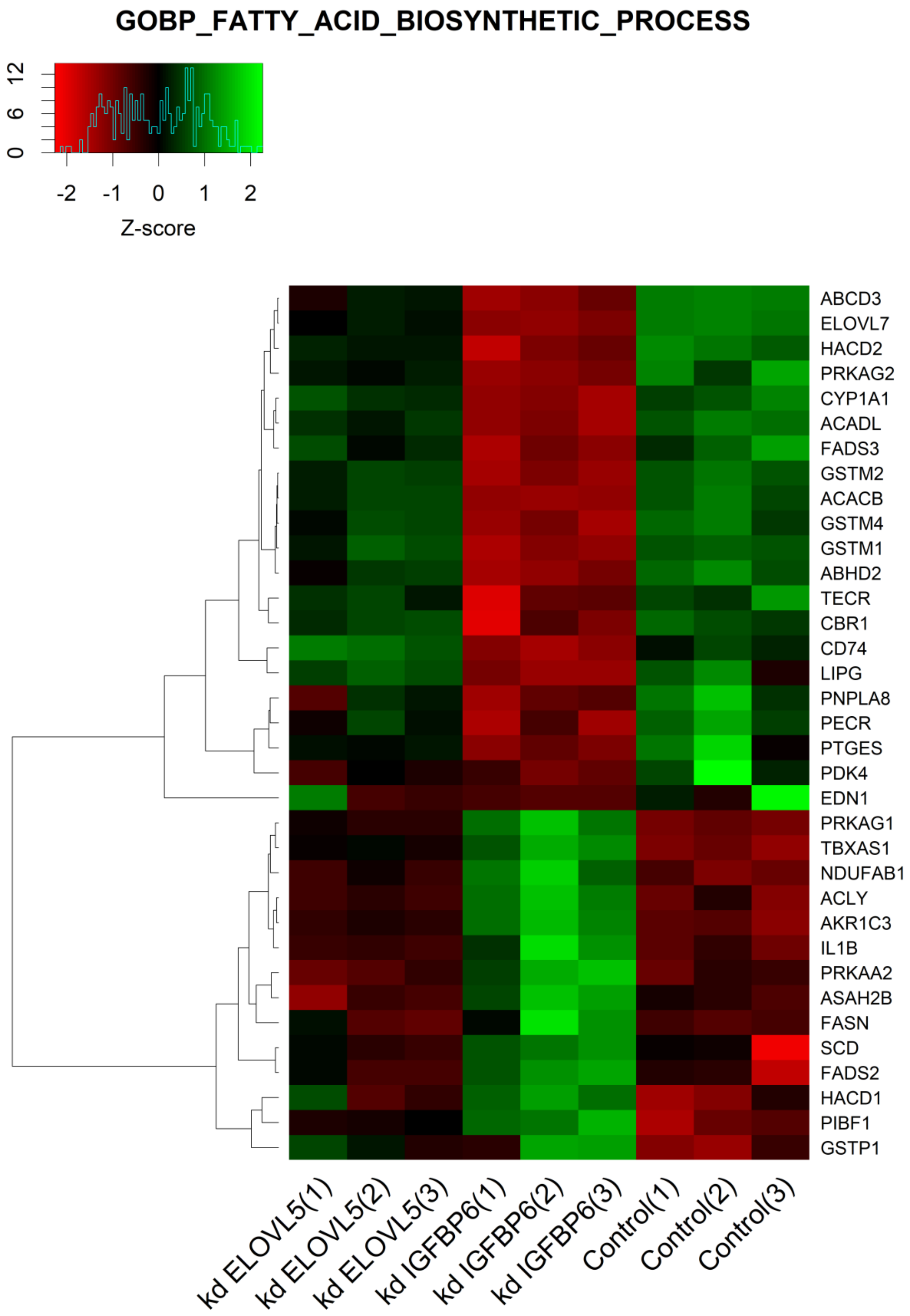

Figure S2. Heatmap of gene expression for "GOBP FATTY ACID BIOSYNTHETIC PROCESS" gene set.

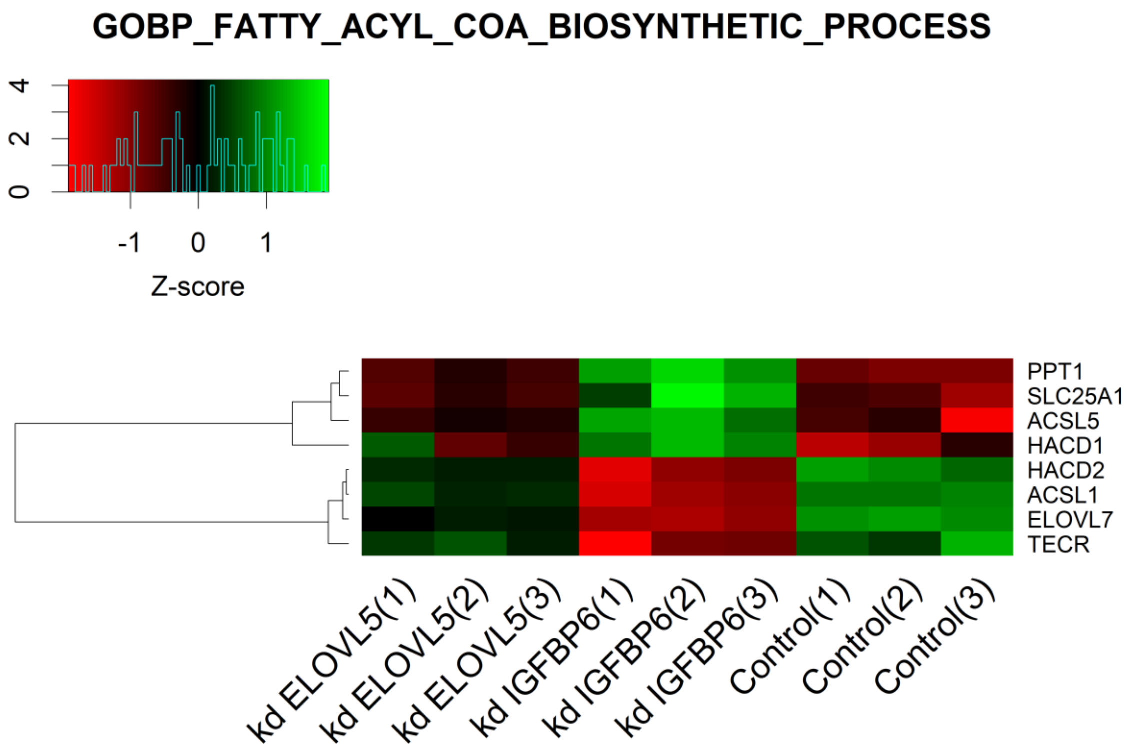

Figure S3. Heatmap of gene expression for "GOBP FATTY ACYL COA BIOSYNTHETIC PROCESS" gene set.

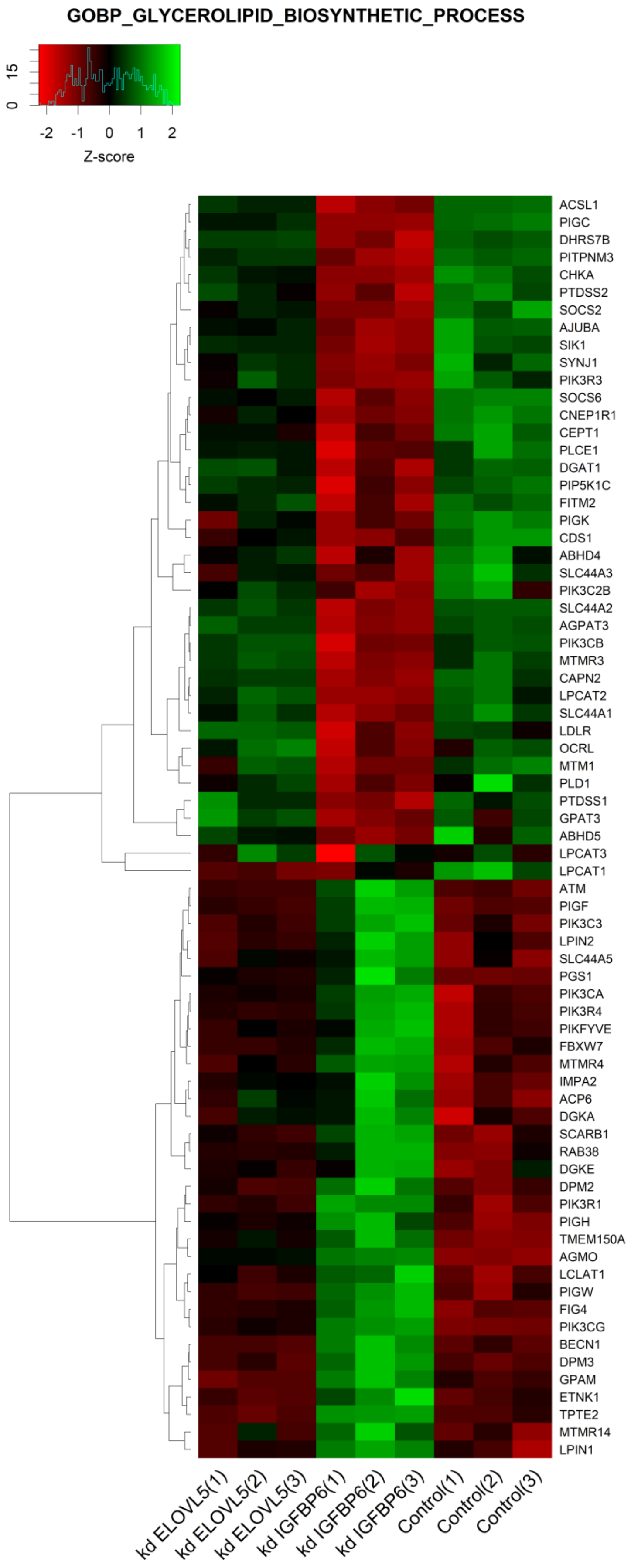

Figure S4. Heatmap of gene expression for "GOBP GLYCEROLIPID BIOSYNTHETIC PROCESS" gene set.

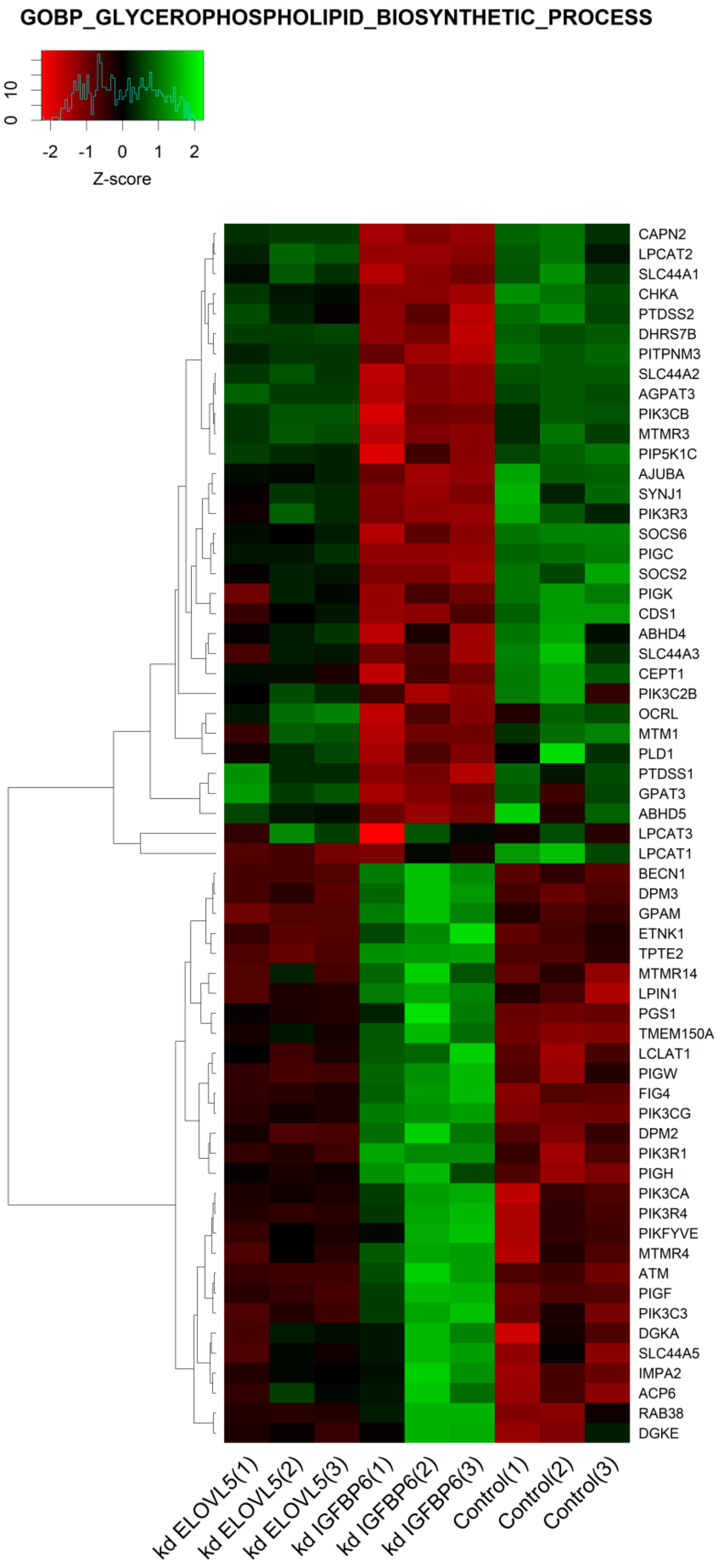

Figure S5. Heatmap of gene expression for "GOBP GLYCEROPHOSPHOLIPID BIOSYNTHETIC PROCESS" gene set.

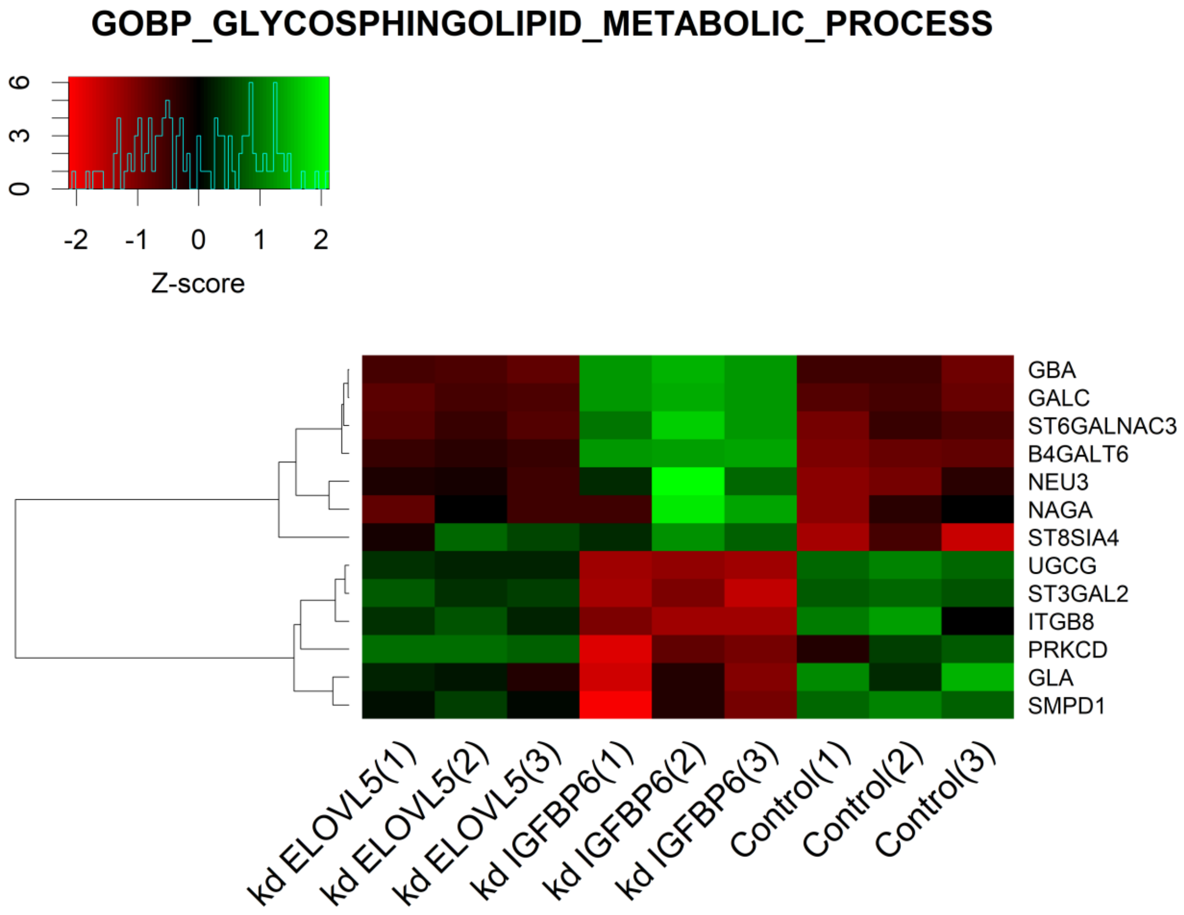

Figure S6. Heatmap of gene expression for "GOBP GLYCOSPHINGOLIPID METABOLIC PROCESS" gene set.

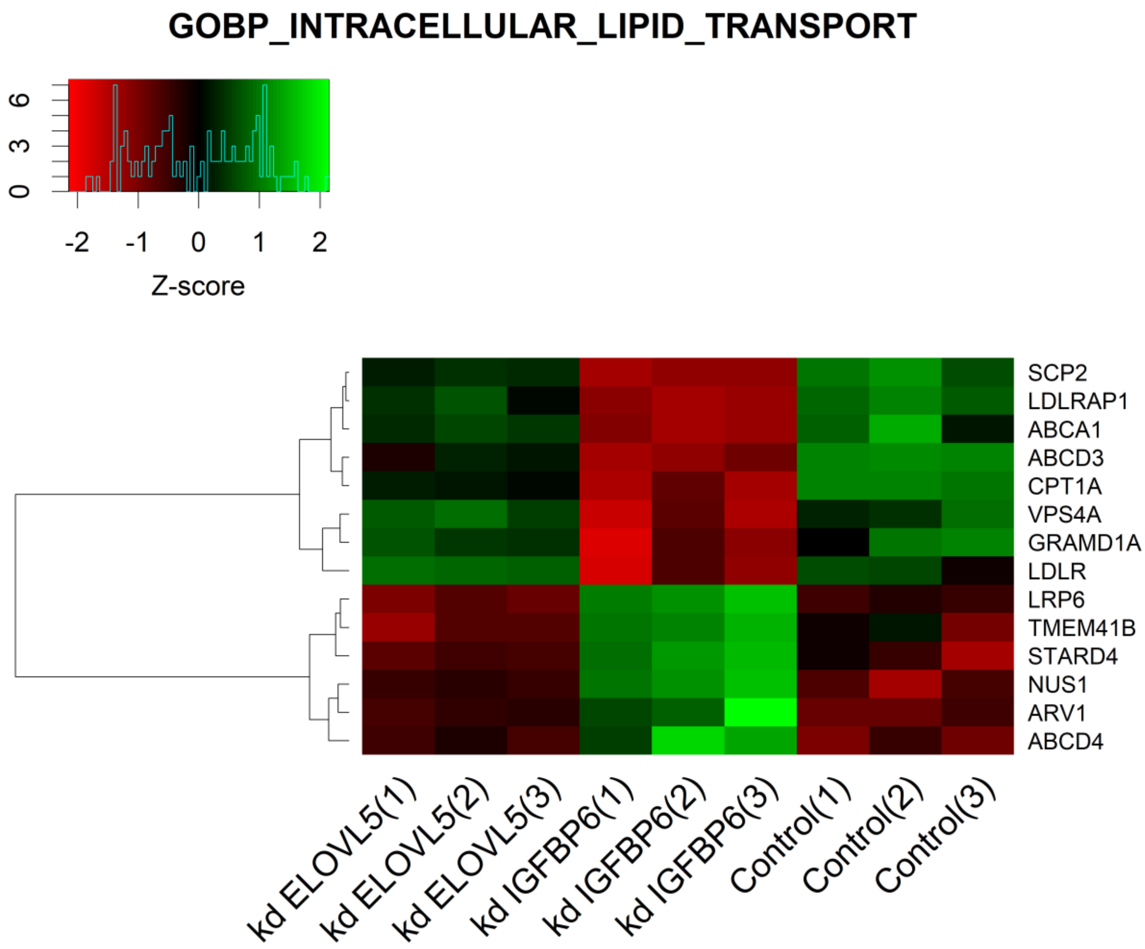

Figure S7. Heatmap of gene expression for "GOBP INTRACELLULAR LIPID TRANSPORT" gene set.

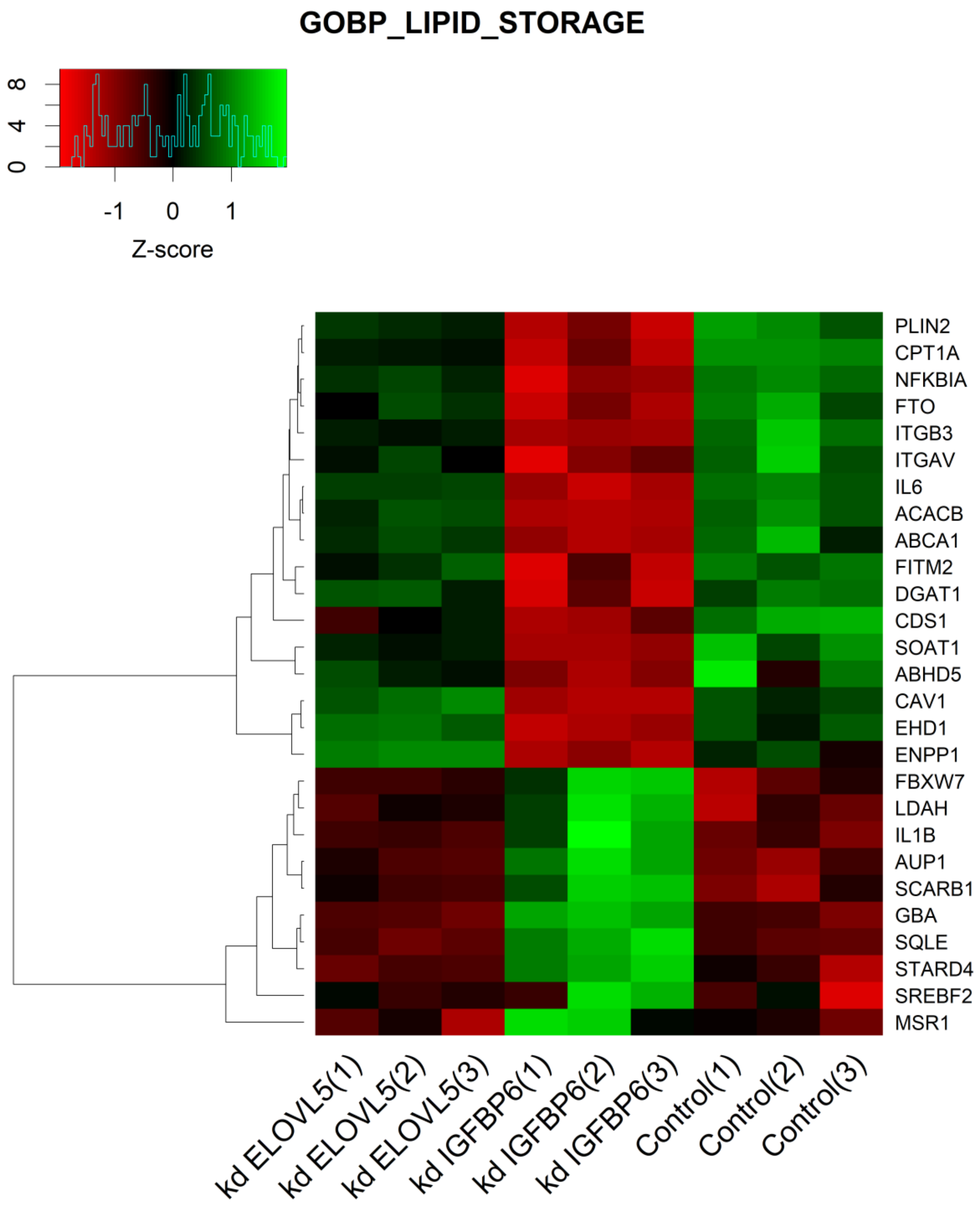

Figure S8. Heatmap of gene expression for “GOBP LIPID STORAGE” gene set.

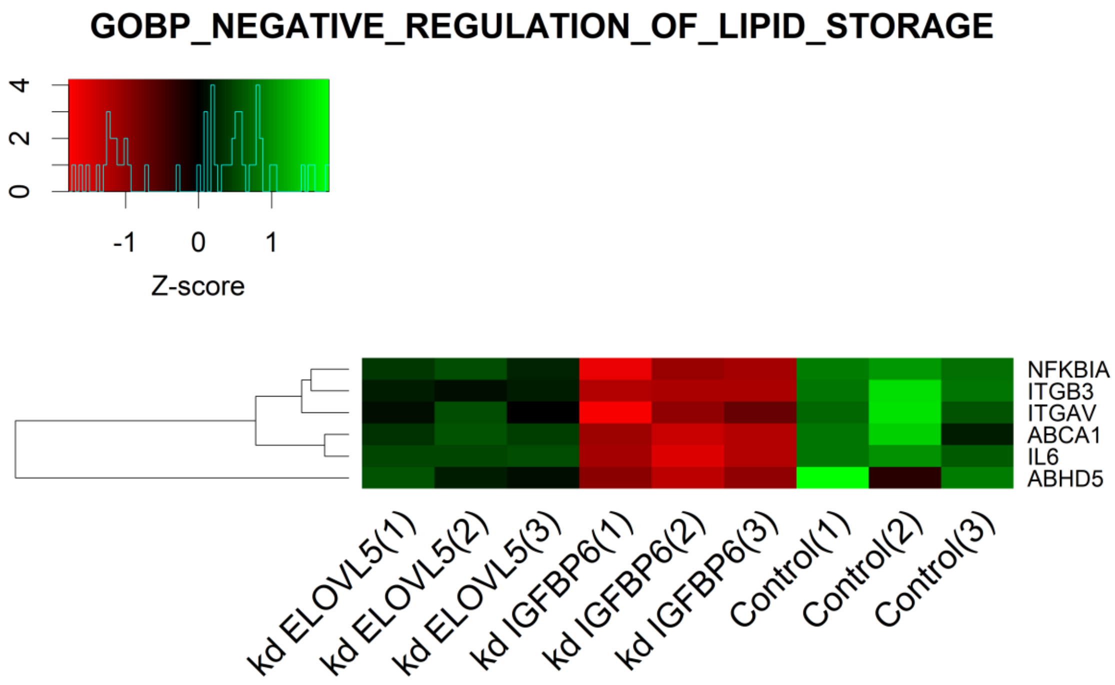

Figure S9. Heatmap of gene expression for "GOBP NEGATIVE REGULATION OF LIPID STORAGE" gene set.

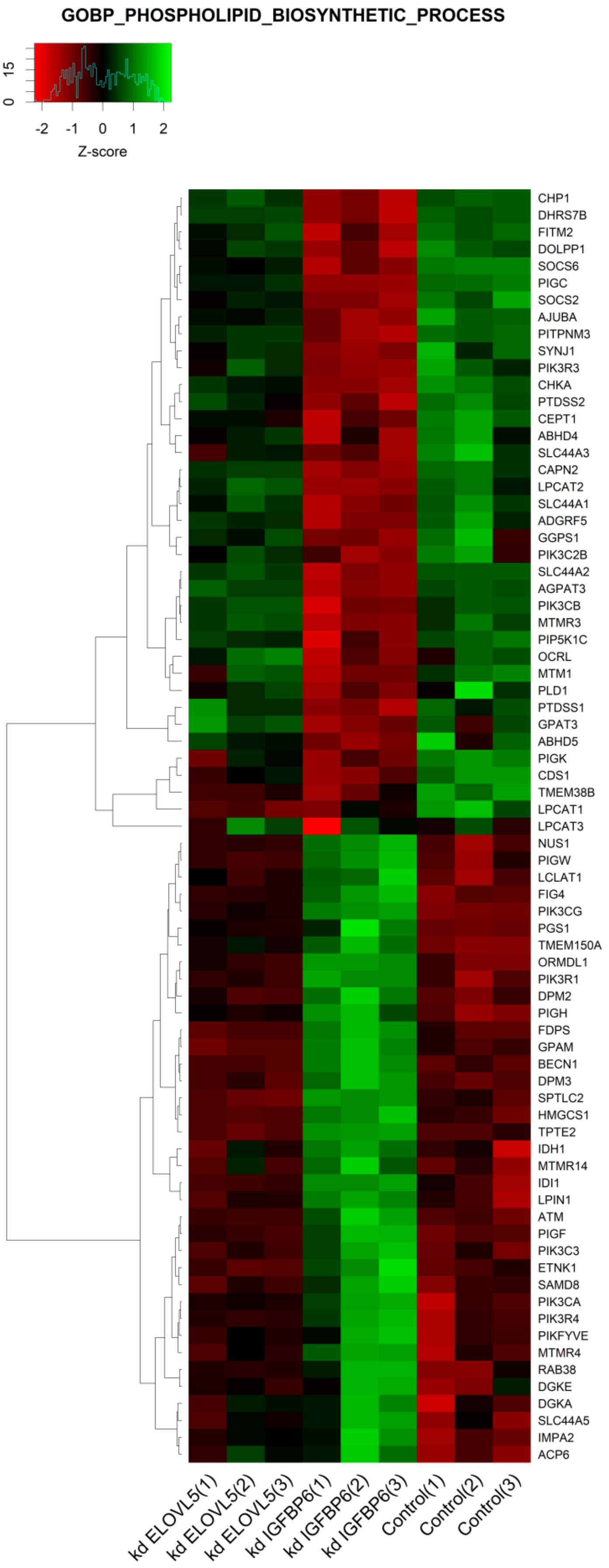

Figure S10. Heatmap of gene expression for “GOBP PHOSPHOLIPID BIOSYNTHETIC PROCESS” gene set.

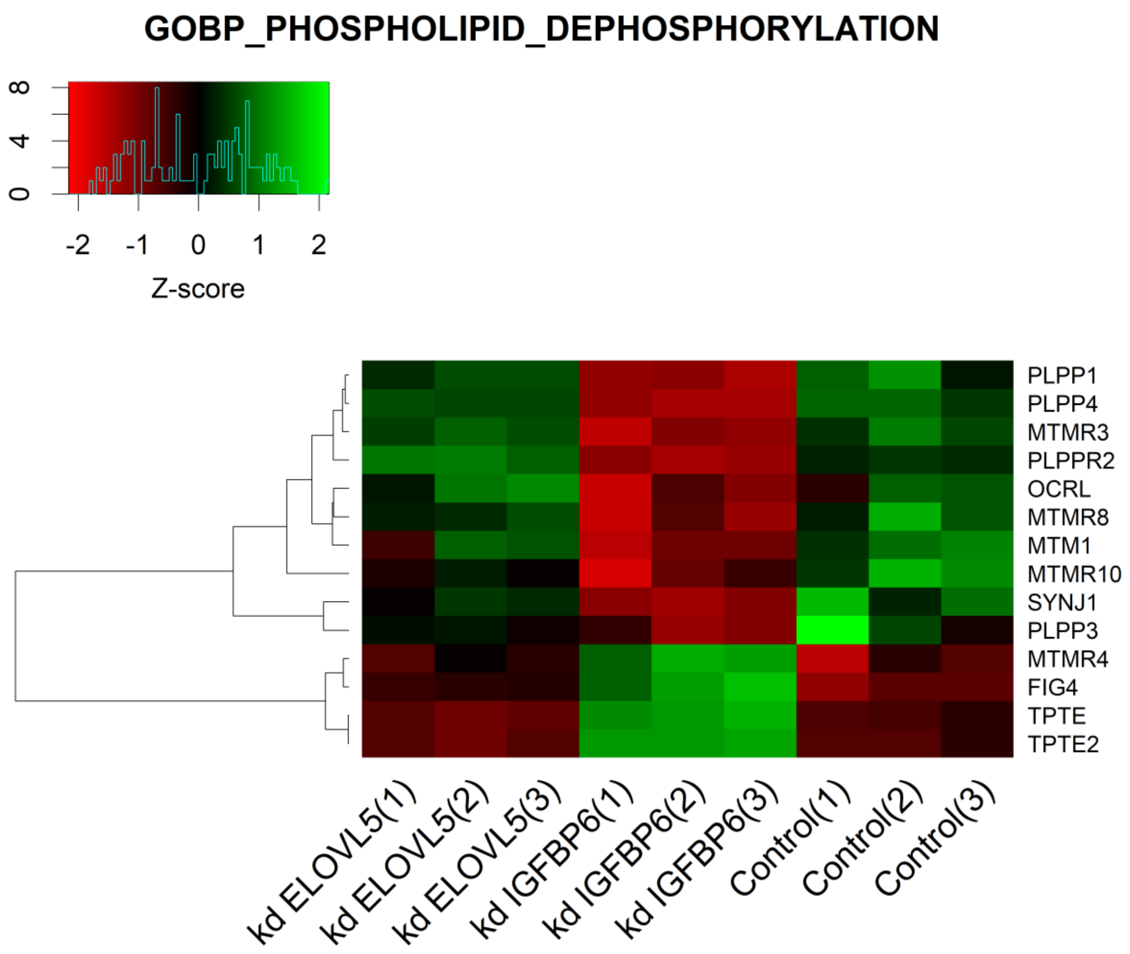

Figure S11. Heatmap of gene expression for "GOBP PHOSPHOLIPID DEPHOSPHORYLATION" gene set.

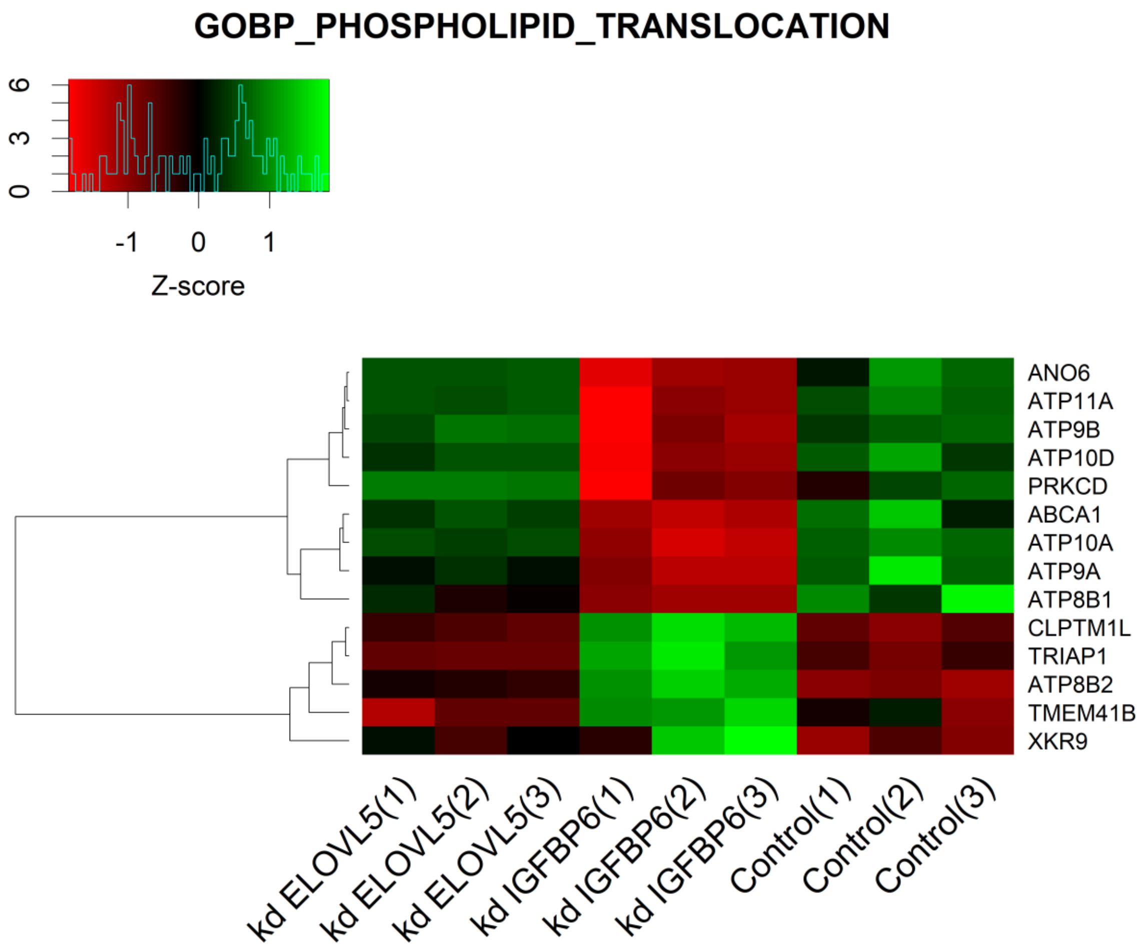

Figure S12. Heatmap of gene expression for “GOBP PHOSPHOLIPID TRANSLOCATION” gene set.

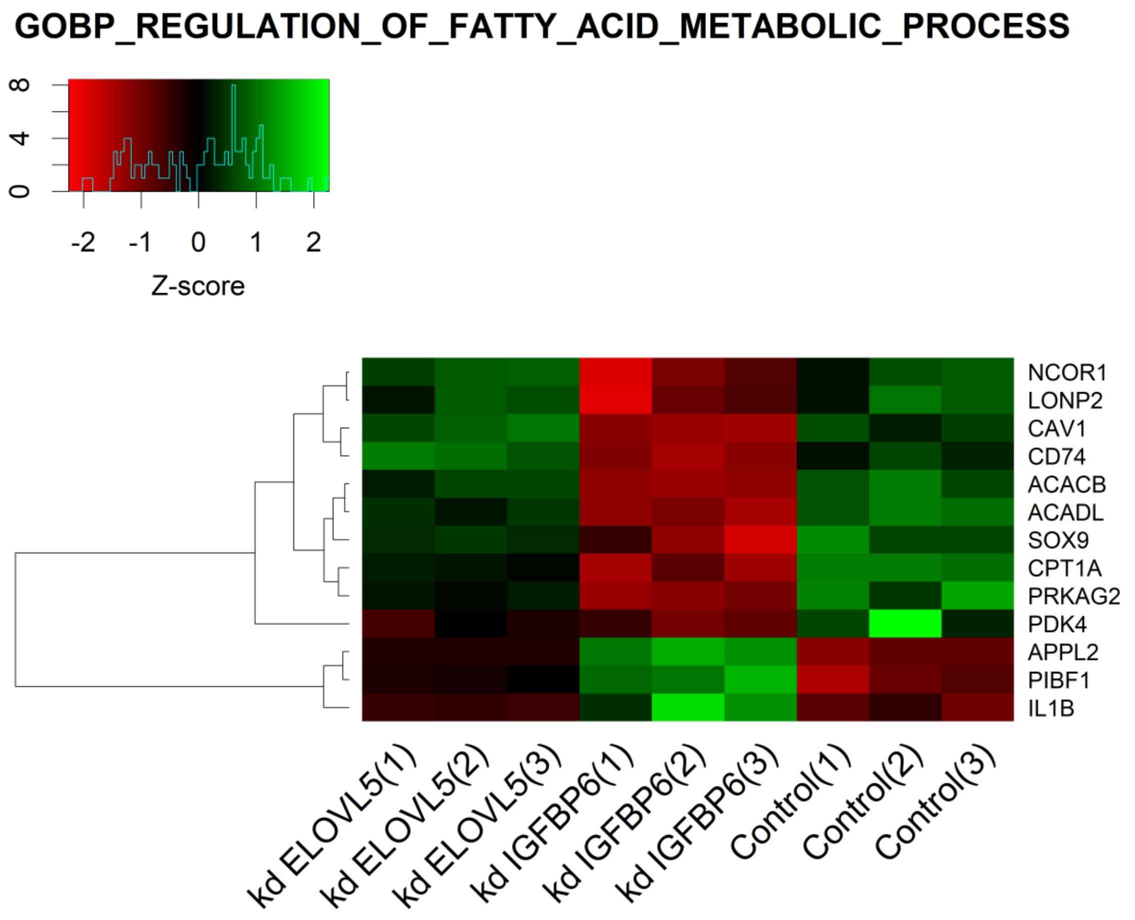

Figure S13. Heatmap of gene expression for "GOBP REGULATION OF FATTY ACID METABOLIC PROCESS" gene set.

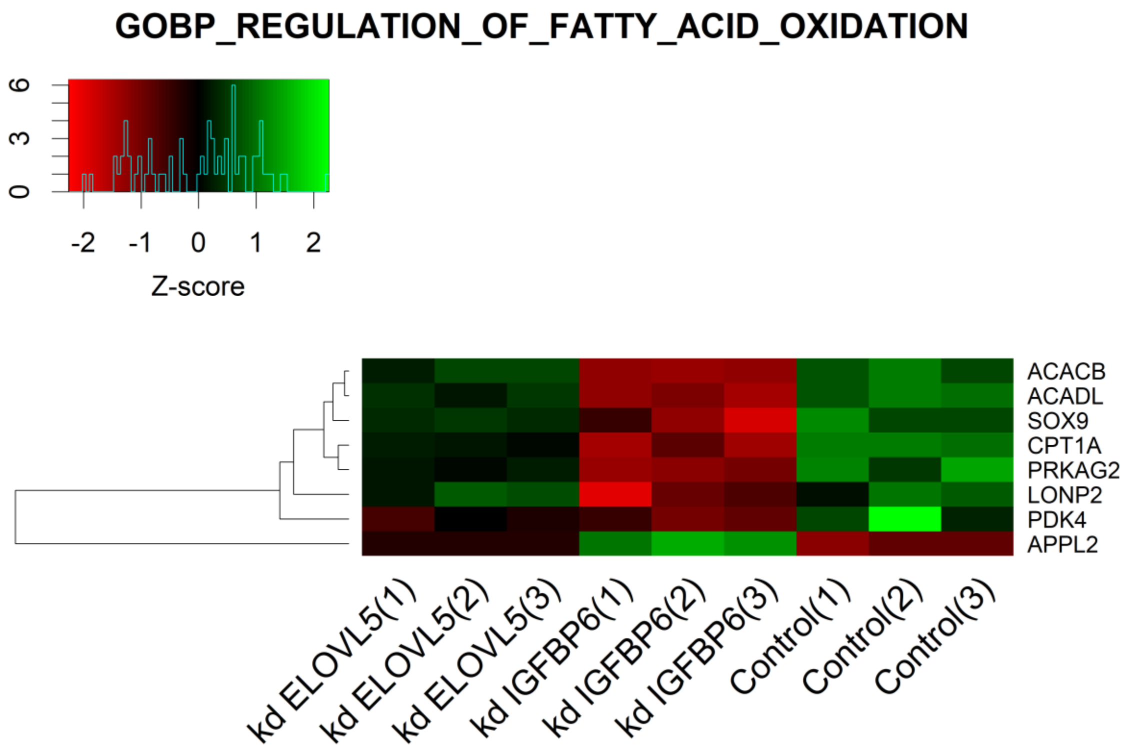

Figure S14. Heatmap of gene expression for "GOBP REGULATION OF FATTY ACID OXIDATION" gene set.

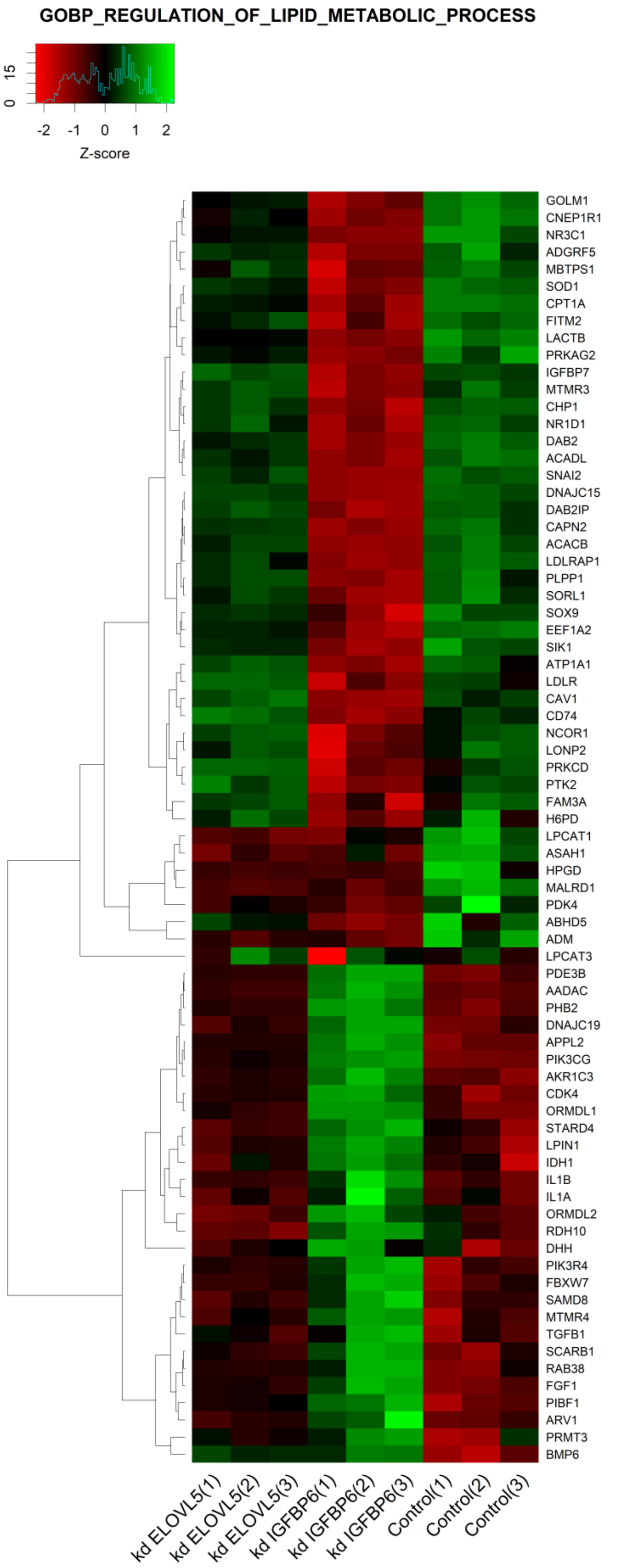

Figure S15. Heatmap of gene expression for “GOBP REGULATION OF LIPID METABOLIC PROCESS” gene set.

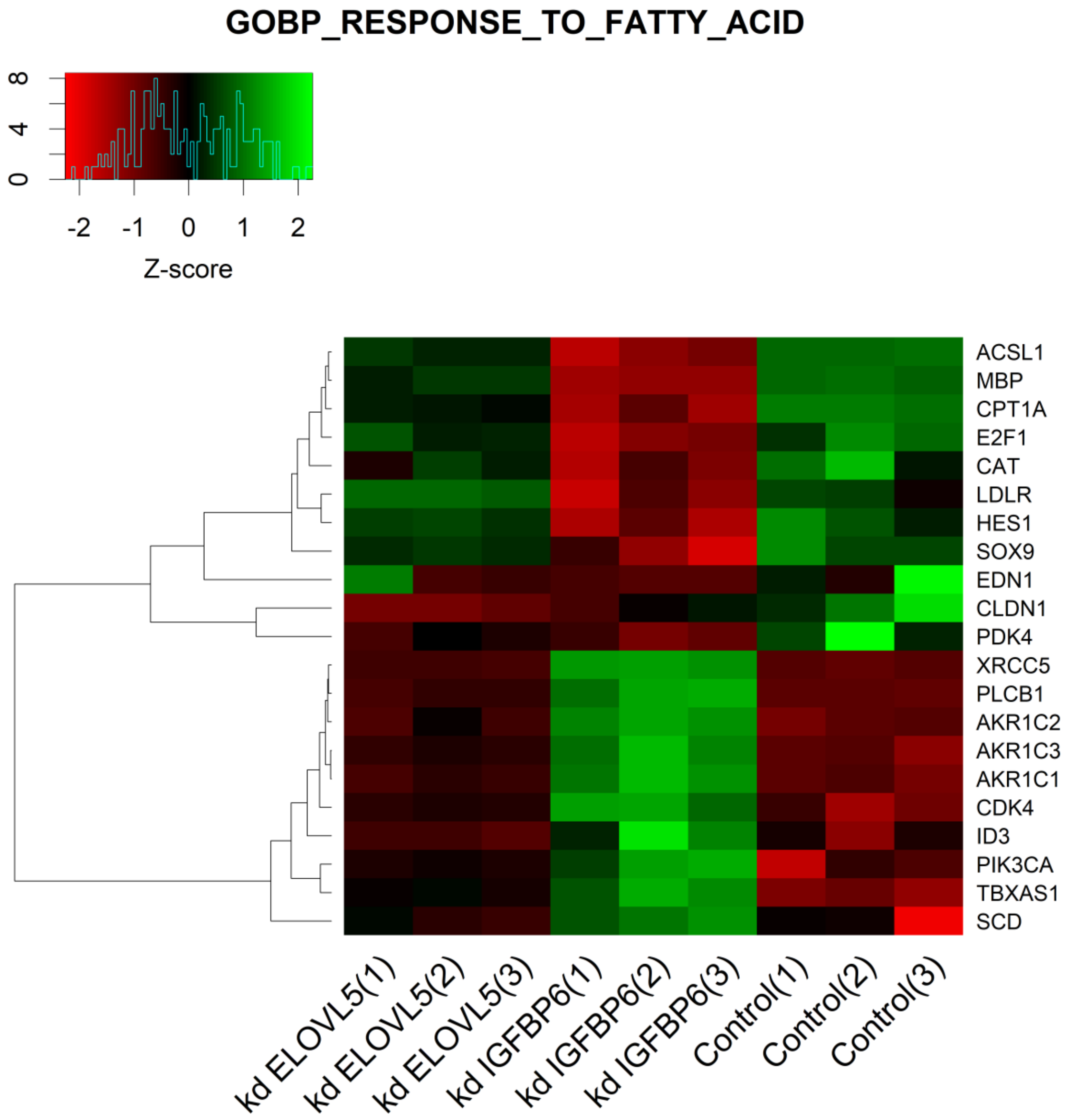

Figure S16. Heatmap of gene expression for "GOBP RESPONSE TO FATTY ACID" gene set.

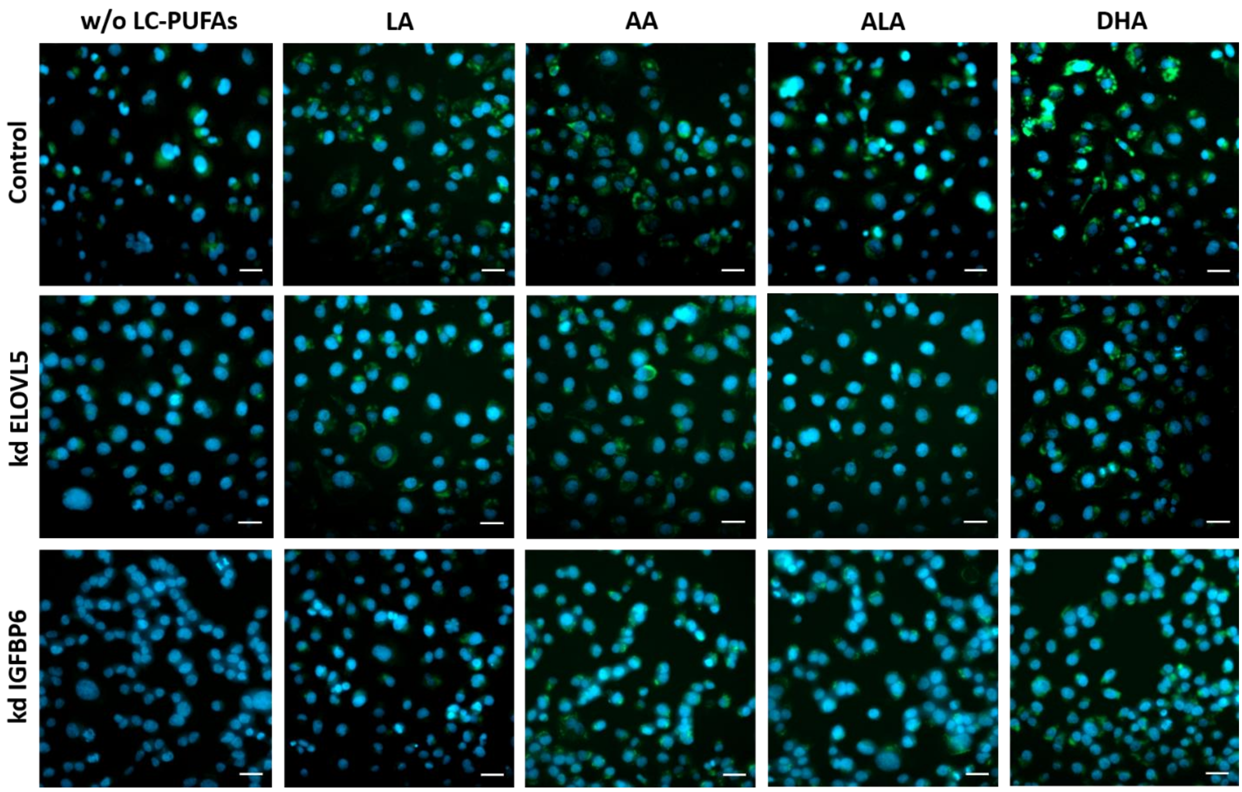

Figure S17. Lipid droplets staining in MDA-MB-231 cells in control medium and in the medium containing 50 µM of LA, AA, ALA and DHA. Scale bars indicate 50 µm. Nuclei (blue) were stained with 4′,6-diamidino-2-phenylindole (DAPI), lipid droplets (green) were stained with BOBIPY.

Table S1. Properties of the utilized breast cancer transcriptomic data sets.

**Dataset**	**Platform**	**Number of samples (Estrogen Receptor status)**	**Dataset ID**
cBioMETABRIC	Illumina HumanHT-12 v3	445(-)	1
		1459(+)	2
GSE102484	Affymetrix HG U133Plus2	283(-)	3
		400(+)	4
GSE12093	Affymetrix HG U133A	136(+)	5
GSE17705	Affymetrix HG U133A	298(+)	6
GSE22220	Illumina humanRef-8 v1_0	82(-)	7
		134(+)	8
GSE3494	Affymetrix HG U133A Affymetrix HG U133B	34(-)	9
		213(+)	10
		4(?)	N/A
GSE58644	Affymetrix HG 1_0 ST	70(-)	11
		250(+)	12
		1(?)	N/A
GSE6532	Affymetrix HG U133Plus2	87(+)	13
	Affymetrix HG U133A Affymetrix HG U133B	45(-)	14
		262(+)	15
		20(?)	N/A
TCGA BRCA	Illumina HiSeq 2000	261(-)	16
		897(+)	17
		63(?)	N/A

Table S2. Oligonucleotide primers for RT-PCR.

Gene	Sequences
FADS1	**Forward:**<br>5'- CCATGCCAAGCCCAACTGCT -3'<br>**Reverse:**<br>5'- TGTGCTGGTGGTTGTACGGC -3'
FADS2	**Forward:**<br>5'- AGCACCACGCCAAGCCTAAC -3'<br>**Reverse:**<br>5'- GCTGCCATTCGCCCAGAACAA -3'
ELOVL7	**Forward:**<br>5'- TGTCCTCGCCTCTGCCACAA -3'<br>**Reverse:**<br>5'- ACCCCAGCCAGACATCACAAAC -3'
EEF1A1	**Forward:**<br>5'-CCCTAAAAGCCAAAATGGGAAA-3'<br>**Reverse:**<br>5'-TAGTGGTGGACTTGCCCGAAT-3'
ACTB	**Forward:**<br>5'- CTGGAACGGTGAAGGTGACA -3'<br>**Reverse:**<br>5'- AAGGGACTTCCTGTAACAACGCA -3'

Table S3. HPLC Gradient Program for analysis of PUFA content in cell culture medium.

Time, min	Phase A (deionized H_2O with 0.1% vol. HCOOH), %	Phase B (Acetonitrile with 0.1% vol. HCOOH), %
0,00	22,5	77,5
5,00	17,5	82,5
5,50	17,5	82,5
6,50	22,5	77,5
7,00	22,5	77,5

Table S4. Changes of fatty acids composition in MDA-MB-231 cells after knockdown of *ELOVL5* and *IGFBP6* genes.

Fatty acid	Knockdown of *ELOVL5*		Knockdown of *IGFBP6*	
	Fold change	*p*-value	Fold change	*p*-value
C14:0	-1.6	0.024	-4.7	0.030
C14:1	-2.2	0.018	-30.4	0.021
C16:0	-1.2	0.097	-1.9	0.002
C16:1n-7	1.1	0.717	-2.2	0.064
C16:2n-7	-1.5	0.512	-4.2	0.061
C18:0	-1.0	0.812	-1.5	0.044
C18:1	1.3	0.512	-1.0	0.925
C18:2	-1.0	0.916	-2.8	0.029
C18:3a	2.8	0.217	-4.8	0.127
C18:3b	1.6	0.287	-3.8	0.055
C18:4n-3	1.5	0.632	3.0	0.081
C20:0	-2.7	0.741	-17.1	0.335
C20:1n-9	-1.4	0.063	-1.1	0.462
C20:2a	-2.8	0.012	-1.9	0.014
C20:2b	-2.0	0.172	-1.9	0.244
C20:3a	-1.3	0.269	-2.7	0.026
C20:3b	1.1	0.740	-1.3	0.100
C20:4	-1.1	0.504	-2.5	0.032
C20:5n-3 (EPA)	1.6	0.064	-6.3	0.005
C22:1n-9	-2.1	0.471	-1.5	0.770
C22:2	-2.2	0.236	-1.2	0.698
C22:3a	-1.8	0.132	-1.6	0.128
C22:3b	-2.1	0.104	1.4	0.294
C22:4n-6	-2.2	0.023	-1.8	0.016
C22:5	-1.3	0.139	-2.3	0.016
C22:6n-3 (DHA)	-1.2	0.055	-3.6	0.001
C24:0	-6.4	0.021	-1152.3	0.009
C24:3a	-2.5	0.495	-2.2	0.457
C24:4a	-1.4	0.348	-1.7	0.307
C24:4b	-3.3	0.214	1.2	0.704
C24:5	-1.5	0.728	-12.7	0.289
C24:6n-3	-1.5	0.122	-2.3	0.029

Table S5. Fitting results (linear models) of the uptake kinetics of PUFAs.

Cells	Fatty acid	Coefficient	Mean	Se	p-value	R2
Control	AA	slope	-0.858	0.050	6.53E-05	0.9868
Control	AA	intercept	31.744	0.734	1.71E-06	0.9868
kd ELOVL5	AA	slope	-2.872	0.266	8.48E-03	0.9831
kd ELOVL5	AA	intercept	30.954	1.337	1.86E-03	0.9831
kd IGFBP6	AA	slope	-1.859	0.175	8.78E-03	0.9825
kd IGFBP6	AA	intercept	32.313	0.881	7.43E-04	0.9825
Control	ALA	slope	-0.776	0.067	3.10E-04	0.9714
Control	ALA	intercept	51.462	0.985	8.05E-07	0.9714
kd ELOVL5	ALA	slope	-1.574	0.093	7.18E-05	0.9862
kd ELOVL5	ALA	intercept	50.492	1.378	3.31E-06	0.9862
kd IGFBP6	ALA	slope	-1.734	0.070	1.59E-05	0.9935
kd IGFBP6	ALA	intercept	51.935	1.038	9.55E-07	0.9935
Control	DHA	slope	-0.304	0.053	4.64E-03	0.8908
Control	DHA	intercept	23.501	0.788	7.52E-06	0.8908
kd ELOVL5	DHA	slope	-0.676	0.189	2.31E-02	0.7623
kd ELOVL5	DHA	intercept	24.396	2.795	9.49E-04	0.7623
kd IGFBP6	DHA	slope	-0.674	0.059	3.36E-04	0.9702
kd IGFBP6	DHA	intercept	23.457	0.873	1.14E-05	0.9702
Control	LA	slope	-2.532	0.791	8.52E-02	0.8368
Control	LA	intercept	63.167	3.972	3.93E-03	0.8368
kd ELOVL5	LA	slope	-4.805	0.459	8.99E-03	0.9821
kd ELOVL5	LA	intercept	64.910	2.305	1.26E-03	0.9821
kd IGFBP6	LA	slope	-2.747	0.433	2.39E-02	0.9527
kd IGFBP6	LA	intercept	65.079	2.176	1.12E-03	0.9527

Table S6. Results of Chi-squared test for flow cytometry data.

Cell	Time	Comparison	Adjusted p-value
Control	3h	LA vs Blank	$< 1 \cdot 10^{-3}$
Control	3h	AA vs Blank	$< 1 \cdot 10^{-3}$
Control	3h	ALA vs Blank	$< 1 \cdot 10^{-3}$
Control	3h	DHA vs Blank	$< 1 \cdot 10^{-3}$
kd ELOVL5	3h	LA vs Blank	$< 1 \cdot 10^{-3}$
kd ELOVL5	3h	AA vs Blank	$< 1 \cdot 10^{-3}$
kd ELOVL5	3h	ALA vs Blank	$< 1 \cdot 10^{-3}$
kd ELOVL5	3h	DHA vs Blank	$< 1 \cdot 10^{-3}$
kd IGFBP6	3h	LA vs Blank	$< 1 \cdot 10^{-3}$
kd IGFBP6	3h	AA vs Blank	$< 1 \cdot 10^{-3}$
kd IGFBP6	3h	ALA vs Blank	$< 1 \cdot 10^{-3}$
kd IGFBP6	3h	DHA vs Blank	$< 1 \cdot 10^{-3}$
Control	20h	LA vs Blank	$< 1 \cdot 10^{-3}$
Control	20h	AA vs Blank	$< 1 \cdot 10^{-3}$
Control	20h	ALA vs Blank	$< 1 \cdot 10^{-3}$
Control	20h	DHA vs Blank	$< 1 \cdot 10^{-3}$
kd ELOVL5	20h	LA vs Blank	$< 1 \cdot 10^{-3}$
kd ELOVL5	20h	AA vs Blank	$< 1 \cdot 10^{-3}$
kd ELOVL5	20h	ALA vs Blank	$< 1 \cdot 10^{-3}$
kd ELOVL5	20h	DHA vs Blank	$< 1 \cdot 10^{-3}$
kd IGFBP6	20h	LA vs Blank	$< 1 \cdot 10^{-3}$
kd IGFBP6	20h	AA vs Blank	$< 1 \cdot 10^{-3}$
kd IGFBP6	20h	ALA vs Blank	$< 1 \cdot 10^{-3}$
kd IGFBP6	20h	DHA vs Blank	$< 1 \cdot 10^{-3}$

Table S7. Significantly changed (FDR p < 0.05) expression of TAG biosynthesis genes after knockdown of *IGFBP6* gene according to the transcriptomic analysis.

**Gene symbol**	**Description**	**Fold change**	**FDR *p*-value**
AGPAT3	1-Acylglycerol-3-Phosphate O-Acyltransferase 3	-1,63	3,43E-08
GPAT2	Glycerol-3-Phosphate Acyltransferase 2, mitochondrial	-1,22	4,10E-03
GPAT3	Glycerol-3-Phosphate Acyltransferase 3	-1,58	3,50E-03
DGAT1	Diacylglycerol O-Acyltransferase 1	-1,7	1,69E-05
